# Cisplatin protects mice from challenge of *Cryptococcus neoformans* by targeting the Prp8 intein

**DOI:** 10.1080/22221751.2019.1625727

**Published:** 2019-06-21

**Authors:** Zhong Li, Bin Fu, Cathleen M. Green, Binbin Liu, Jing Zhang, Yuekun Lang, Sudha Chaturvedi, Marlene Belfort, Guojian Liao, Hongmin Li

**Affiliations:** a New York State Department of Health, Wadsworth Center, Albany, NY, USA; b College of Pharmaceutical Sciences, Southwest University, Chongqing, People’s Republic of China; c Department of Biological Sciences and RNA Institute, University at Albany, Albany, NY, USA; d Department of Biomedical Sciences, School of Public Health, University at Albany, Albany, NY, USA

**Keywords:** Cisplatin, antifungal, *Cryptococcus*, Prp8 intein, mouse model

## Abstract

The Prp8 intein is one of the most widespread eukaryotic inteins, present in important pathogenic fungi, including *Cryptococcus* and *Aspergillus* species. Because the processed Prp8 carries out essential and non-redundant cellular functions, a Prp8 intein inhibitor is a mechanistically novel antifungal agent. In this report, we demonstrated that cisplatin, an FDA-approved cancer drug, significantly arrested growth of Prp8 intein-containing fungi *C. neoformans and C. gattii,* but only poorly inhibited growth of intein-free *Candida* species. These results suggest that cisplatin arrests fungal growth through specific inhibition of the Prp8 intein. Cisplatin was also found to significantly inhibit growth of *C. neoformans* in a mouse model. Our results further showed that cisplatin inhibited Prp8 intein splicing *in vitro* in a dose-dependent manner by direct binding to the Prp8 intein. Crystal structures of the apo- and cisplatin-bound Prp8 inteins revealed that two degenerate cisplatin molecules bind at the intein active site. Mutation of the splicing-site residues led to loss of cisplatin binding, as well as impairment of intein splicing. Finally, we found that overexpression of the Prp8 intein in cryptococcal species conferred cisplatin resistance. Overall, these results indicate that the Prp8 intein is a novel antifungal target worth further investigation.

## Introduction

Invasive fungal infections (IFI) remain a major public health challenge. Globally, over 300 million people are affected by IFI, with over 1 million estimated deaths every year [1–4]. IFI are largely caused by species of *Candida*, *Cryptococcus*, and *Aspergillus*. Among these pathogens, *Cryptococcus neoformans* (*Cne*) *and C. gattii* (*Cga*) cause pulmonary cryptococcosis and cryptococcal meningitis (CM), which are extremely difficult to treat, leading to a large burden of morbidity and mortality. Annually, there are over 1 million cases of CM worldwide, with deaths estimated at 700,000 per year [1, 5, 6]. CM is most common in immunocompromised patients, such as those with HIV, severe combined immunodeficiency, or post organ-transplant [7, 8]. However, recent outbreaks caused by *Cga* in non-immunocompromised populations have raised concerns about the overall threat of *Cryptococcus* species to public health [9–11]. Additionally, *Aspergillus fumigatus* (*Afu*)*,* a saprotroph widespread in nature, is one of the most common species to cause aspergillosis in individuals with immunodeficiency. Worldwide, around 600,000 deaths annually are due to various forms of aspergillosis [12]. IFI treatment is usually achieved with combination therapy over a lengthy period of time, ranging from 12–14 weeks to life-long [13, 14]. Chronic treatments for IFI have created a severe drug resistance problem [15–17], therefore, novel drugs with innovative targets are urgently needed.

*Cryptococcus, Aspergillus, Histoplasma, and Blastomyces* fungi, among several other microbial pathogens, contain protein self-splicing elements called inteins. Inteins are *in*ternal pro*tein*s that self-excise from their host proteins and catalyse ligation of the flanking sequences (called exteins) with a natural peptide bond [18–21]. Because inteins frequently interrupt highly conserved sequences of host proteins, they often disrupt functions that are essential for the pathogen’s survival [22]. Therefore, inteins are attractive potential drug targets [22–25]. Cisplatin and a number of platinum-containing small molecules were recently identified as potent inhibitors against the RecA intein from *Mycobacterium tuberculosis* [24, 25]. Furthermore, cisplatin has also been shown to inhibit the growth of *M. tuberculosis* by intein targeting [24].

The fungal intein of interest here interrupts the gene encoding pre-mRNA processing factor 8 (Prp8) [26–29], which is the largest, most conserved, and highly essential protein of the spliceosome responsible for mRNA splicing. The Prp8 intein belongs to the class 1 intein family [26, 28, 29]. Structural and sequence analyses indicate that the Prp8 intein is most commonly located at an area on the surface of the Prp8 host protein responsible for binding of U2 snRNA, U6 snRNA, and intron RNA [30, 31]. This suggests that an unspliced Prp8 intein likely would interfere with the binding of these RNA elements to the spliceosome, and thus, disrupt spliceosome function.

To explore the potential of the Prp8 intein as a therapeutic target, we developed a split luciferase-based Prp8 intein splicing assay. This revealed that cisplatin, an FDA-approved chemotherapy drug, is a potent inhibitor of Prp8 intein splicing. We demonstrated that cisplatin can inhibit growth of the Prp8 intein-containing *C. neoformans* and *C. gattii*, but not inteinless *Candida albicans*. Moreover, cisplatin treatment can significantly protect mice from challenge of *C. neoformans in vivo*. Furthermore, we used biochemistry, mass spectrometry, and solved a crystal structure to demonstrate that cisplatin targets the Prp8 intein as the mechanism for inhibition of fungal growth. These results validate the Prp8 intein as a viable target for development of small molecule drugs against IFI.

## Materials and methods

### Ethics statement

All animal studies were approved by the Animal welfare and Research Ethics Committee at College of Pharmaceutical Sciences of Southwest university (Approval ID: 20160922). All procedures were conducted in accordance with the Guide for the Care and Use of Laboratory Animals of China.

### Compounds

Tris(2-carboxyethyl)phosphine (TCEP) and cisplatin were from Sigma-Aldrich.

### Cloning, expression and purification

The split luciferase-Prp8 was constructed using a mega-PCR mutagenesis approach [32]. First, the full-length (FL) RLuc (amino acids 1-311) was cloned into the pET26b vector using the mega-PCR approach [33], with a pair of primers, RLuc_N_P26_F and RLuc_C_P26_R (Supplemental Table 2). The FL RLuc construct FLuc-pET26b was verified by DNA sequencing. To generate a Prp8 splicing luciferase construct, we inserted a His-tag between Prp8 residues 133 and 134. The rationale to insert the His-tag into the Prp8 intein instead of luciferase is to avoid co-purification of reconstituted luciferase due to Prp8 intein splicing activity, which may generate a high background signal. A DNA fragment was generated representing the RLuc-Prp8 with His-tag inserted between the Prp8 aa 133 and 134, using an overlapping strategy with pairs of primers (Prp8_N_Rluc229_F and Prp8_addHis_R for aa 1-133; Prp8_addHis_F and PRP8_C_Rluc231_R for aa 134-311). The overlapping PCR product was used as a megaprimer for PCR mutagenesis with the FLuc-pET26b plasmid as a template. The PCR product was digested with *DpnI* and transformed into DH5α *Escherichia coli*. Clones were verified by DNA plasmid sequencing.

The *Cga* MIG Prp8 construct was made by amplifying the Prp8 intein with primer pair IDT3977/3978, including 5 native N-extein (FWEKA) and C-extein (SGFEE) residues, from *C. gattii* NIH444 genomic DNA. Internal SOE PCR [34] was performed to remove an internal ClaI cut site from the intein using the primers IDT3995/3996 listed in Supplemental Table 2. The full SOE PCR product was digested with SphI and ClaI and inserted into pACYC Duet backbone. Ligation was performed with T4 DNA ligase (New England Biolabs) and transformed into DH5α. Clones were verified by DNA sequencing (Eton Biosciences).

Two constructs were made to express the *Cga* Prp8 intein (pET28a and pXI). For pXI, the *Cga* Prp8 intein was amplified from genomic DNA of *C. gattii* NIH444 plus three native N-extein residues (EKA) using primer pair IDT4283/4284 (Supplemental Table 2). Using InFusion technology (Clontech), including 15 bp overhang to the destination vector, the undigested insert was cloned into the pXI vector [35] at the EcoRI and HindIII sites, creating a chitin binding domain (CBD)-Prp8 intein fusion protein. Similarly, the same intein was amplified with InFusion primer pair IDT4038/4039 to pET28a at the HindIII and NotI sites. Clones were verified by DNA sequencing (Eton Biosciences). The Prp8 mutants (C1A, H62A, D95A, and H169A) in the pXI and MIG vectors were generated using the Strategene point mutagenesis approach with the primers as shown in Supplemental Table 2. All constructs were verified by sequencing.

The Flag-Prp8 intein construct was cloned into the pXL1-PTEF1 vector using a similar megaprimer strategy with primers listed in Supplemental Table 2. Three fragments were first amplified, encoding the Prp8 1–132 (primers: pXL1_Cgaprp8_N_F and cgaPr8_A132_Flag3x_R; template, Cga_Prp8), 3x Flag-tag (primers: cgaPrp8_A132_Flag3x_F and flag3x_Cgaprp8_T133_R; template, an in-house 3x Flag-tag vector), and Prp8 133–170 (primers: flag3x_Cgaprp8_T133_F and pXL1_CgaPr8_C-R; template, Cga_prp8), respectively. The three PCR fragments were mixed and re-amplified using the pXL1_Cgaprp8_N_F and pXL1_CgaPr8_C-R primers to obtain the FL Flag-tagged Prp8, which was used as a megaprimer for PCR mutagenesis with the pXL1-PTEF1 plasmid as a template to generate the Flag-Prp8 expression vector under the control of the strong promoter TEF1. The Flag-AAAA mutant (C1A/H62A/D95A/H169A) was synthesized by Gene Universal and ligated into the pXL1-PTEF1 vector using the FseI and PacI restriction sites. The sequence-verified Flag-Prp8 WT and mutant plasmids were transformed into the *Crytococcus gattii* NIH444 strain for functional studies, using a Bio-Rad electroporator.

All proteins were expressed in *E. coli* strain BL21(DE3) (EMD Biosciences) at 16°C overnight. The Prp8 intein (pET28a) was purified through a nickel-nitrilotriacetic acid column (Qiagen). The Prp8 intein WT and mutants (C1A, H62A, D95A, and H169A) in the pXI vector were purified through a chitin bead column, as described [36]. To generate seleno-methionine (Se-Met) substituted Prp8 intein, the Prp8 intein (pET28a) was transformed into B834(DE3) cells and cultured in minimal media supplemented with Se-Met, as we described previously [37]. All samples were finally purified by a gel filtration 16/60 Superdex 200 column (GE HealthCare).

### The split luciferase Prp8 intein splicing assay

The purified RLuc-Prp8 fusion protein was diluted into the intein splicing buffer (20 mM Tris pH 8.0, 100 mM NaCl, 5% Glycerol, and 0.05% CHAPS) and incubated with cisplatin or DMSO control for 30 min. The TCEP was added to a final concentration of 100 µM. The reactions were incubated at room temperature overnight. Upon addition of RLuc substrate coelenterazine, the Prp8 intein splicing was monitored using a Veritas luminometer for the RLuc construct.

### The MIG Prp8 splicing assay

The MIG Prp8 splicing assay was carried out as described previously [38]. Briefly, the WT and all the mutant plasmids were transformed into MC1061 cells. A 5 ml overnight culture of a single colony for each construct was inoculated into 250 ml fresh medium and grown at 37°C with 250 rpm shaking to an OD_600_ of 0.6–0.8. Cells were induced with 0.5 mM IPTG for 2 h at 37°C. Cells were harvested by centrifugation and immediately stored at −80°C. Cell pellets were resuspended in a buffer containing 50 mM Tris, pH 8.0, and 150 mM NaCl and then sonicated. The supernatants were stored at −80°C after centrifugation.

For the MIG Prp8 splicing assay, the frozen aliquot was considered as time 0. Aliquots of 20 µl of each sample proteins (WT or mutant) were added to an Eppendorf tube. For the cisplatin inhibition experiment, serial dilutions of cisplatin were added to the tube, and incubated at room temperature for 1 h. TCEP was added to a final concentration of 100 µM. The mixtures were incubated overnight (∼18 h) and then analysed in SDS-PAGE without boiling. For the WT and mutant splicing assays, the samples were incubated at 22°C or 37°C, with or without 100 µM TCEP, for 18 h. The samples were analysed in SDS-PAGE without boiling. The gels were quantified using green fluorescent protein (GFP) fluorescence by the Bio-Rad Gel Doc EZ.

### Native polyacrylamide gel electrophoresis assay

The gel mobility shift assays were carried out as described previously [25]. Briefly, WT or mutant proteins at 30 µM were incubated with or without 300 µM cisplatin overnight in a buffer containing 50 mM sodium phosphate and 100 mM NaCl, pH 7.0, in the presence or absence of 200 μM TCEP at 25°C for 18 h in the dark. The mixtures were analysed using native polyacrylamide gel electrophoresis (12%). Gels were run under a constant voltage of 150 V for a minimum of 2 h to obtain optimal separation. Bands were visualized using Coomassie Brilliant Blue staining, and quantified using the Bio-Rad GelDoc EZ.

### Electrospray ionization mass spectrometry

The ESI-MS experiment was carried out as describe previously [25]. Briefly, purified *Cga* Prp8 intein (30 µM) in binding buffer (50 mM sodium phosphate, 100 mM NaCl, pH 7.0) was incubated with cisplatin at various concentrations with or without 100 µM or 2 mM TCEP. The incubation was carried out for ∼18 h in the dark. Samples were buffer exchanged into 150 mM ammonium acetate through desalting columns and concentrated using 3 kDa Amicon Ultra centrifugal filter units (EmdMillipore, Bellerica, MA). Mass spectrometry analysis of the protein was carried out on an LTQ Orbitrap Velos instrument (Thermo Scientific, Grand Island, NY) in the positive ion mode.

### Fungal inhibition assay

The fungi were grown overnight in the yeast extract–peptone–dextrose (YPD) medium. The fungi were diluted to a final cell density at OD_530_ of 0.1. Then 20 µl diluted cells were further diluted into 10 ml YPD as a stock. Prior to cell addition, a 96-well plate was prepared with twofold dilution series of compounds (100 µl) at 2× final concentration or DMSO control in YPD. The stock fungi cells (100 µl) were dispensed into the compound-ready 96-well plate. The fungi (treated or DMSO) were incubated at 30°C for 48 h (*Cne or Cga*) or 24 h (*Cal*). The cell cultures were visualized under a microscope. For MIC determination, 20 µl of a 10-fold serial dilution of fungal culture was spread onto YPD Agar plate, followed by incubation at 30°C for 48 h. Colony forming unit (CFU) was counted and non-linear regression of the resulting data was used to calculate MIC_50_ and MIC_90_.

For growth kinetics studies, an empty pXL1-PTEF1 vector, the Prp8-pXL1-PTEF1 (WT Prp8) vector, or the AAAA mutant were electroporated into the *Cga* NIH444 strain. Transformed *Cga* were plated onto YPD plates with G418 (100 µg/ml). A single colony was selected for growth kinetics and inhibition analysis, in the presence or absence of 1.9 µM cisplatin. The culture was monitored by OD_550_ using an EL808 microplate reader at 24, 48, and 72 h.

#### Crystallization, data collection, structure determination, and refinement

For crystallization, the purified and Se-Met-substituted *Cga* Prp8 (pET28a) intein was concentrated to 17.5 mg/ml in a buffer composed of 25 mM Tris, pH 8.5, 150 mM NaCl, and 2 mM DTT. Initial crystallization conditions were obtained by screening the Hampton crystallization screens (I, II, and Research Index HT) using the hanging-drop vapour diffusion method. Large crystals were obtained by mixing 1 μl of intein and 1 μl of reservoir solution containing 1.5 M sodium citrate, 0.1 M Hepes, pH 7.5, 2% isopropanol, and 10 mM DTT. To obtain the Prp8 intein-cisplatin complex, the apo crystals were soaked overnight in the crystallization mother liquor supplemented with 3 mM cisplatin dissolved in DMSO. All crystals were harvested in a cryo-solution containing crystallization mother liquor supplemented with 25% glycerol, and flash-cooled in liquid nitrogen. Diffraction data for the apo crystal was collected to 2.6 Å resolution at 100 K using a Pilatus 6M detector at the BL9-2 beamline of the Stanford Synchrotron Radiation Lightsource (SSRL). Diffraction data for the cisplatin-Prp8 complex crystal were collected with a Mar 325 CCD detector at the SSRL BL14-1 beamline. All diffraction data were processed and scaled using the HKL2000 package [39].

The structure of the apo form of the *Cga* Prp8 was determined by the multiple anomalous diffraction method using the PHENIX program suite [40]. The Prp8-cisplatin complex was determined by Fourier transformation, using the apo Prp8 intein as the starting model. Structural refinement was carried out using PHENIX Refine. Model rebuilding was carried out using Coot [41].

### Mouse model

The *in vivo* efficacy of cisplatin was determined in a murine model of disseminated cryptococcosis. Briefly, groups of six 4–6 week-old female BALB/c mice weighing approximately 20 g were challenged intranasally with *C. neoformans* H99 at 1×10^7^ CFU/mouse. Mice were treated with cisplatin (Hansoh Pharma, China) or control (saline) once daily for 4 days at 24 h post-infection. At day 4 after challenge, mice were euthanized and both lungs were aseptically collected, weighed, homogenized in sterile saline, serially diluted, and plated onto YPD agar. Plates were incubated at 37°C for 48 h and then colonies were enumerated. *Cryptococcus* burden (CFU/g lung) in cisplatin-treated groups was compared to that in the relevant saline control. Significance was determined using the student’s paired two-tailed *t* test. The isolated lung tissues were also fixed in 10% formalin solution and were stained with Haematoxylin Eosin (H&E) for histopathological examination under a light microscope.

### Statistical analysis

All experiments were performed in triplicate unless specified otherwise. GFP fluorescence in gel was quantified using the Bio-Rad Gel Doc EZ system and Image Lab^TM^ software #1709690 (Bio-Rad). One-way ANOVA was used to carry out statistical analyses with the Prism software.

## Results

### Inhibition of the Prp8 intein-containing fungi by cisplatin

Previous research has considered targeting inteins in critical proteins for novel drug development [22, 25]. These studies showed that cisplatin is a potent inhibitor against a mycobacterial intein in a recombinase protein [24, 25]. Because the fungal cryptococcal species *Cne* and *Cga* encode an intein in the essential Prp8 protein, and these inteins belong to the same intein class as the mycobacterial intein, we speculated that cisplatin might also be an inhibitor of the Prp8 intein.

To test this hypothesis, we first investigated whether cisplatin inhibited the growth of Prp8 intein-containing fungi *in vitro* in a cell culture setting. We performed a fungal inhibition microdilution assay [42] and found that cisplatin is a potent inhibitor of representative Prp8 intein-containing *Cne* strains and *Cga* strains (Table 1). All strains were inhibited by cisplatin at MIC_50_ and MIC_90_ levels of 20 µg/ml or less (Table 1). In contrast, cisplatin did not inhibit growth of *C. albicans (Cal*), an intein-free pathogenic fungus, at the MIC_90_ level until a much higher concentration (100 µg/ml). These results are consistent with previous findings that cisplatin inhibited *Cal* with MIC in the range of 100–800 µg/ml [43]. Overall, these results indicate that cisplatin selectively kills Prp8 intein-containing fungi, suggesting the Prp8 intein as a potential drug target for Prp8 intein-containing fungi.

**Table 1.** Inhibition of pathogenic fungi by cisplatin using microdilution assay.

	Strain	Sub-group	MIC_50_ (µg/ml)	MIC_90_ (µg/ml)
*C. neoformans*	NIH H99	VN I	0.92	4.5
	WM148	VN I	2.0	18.0
	WM626	VN II	1.4	9.0
*C. Gattii*	NIH444	VG IIa	1.11	8.1
	WM276	VG I	1.9	20.0
	CA1222	VG IIIa	1.5	13.5
	VM779	VG IV	1.5	14
*C. Albicans*	ATCC90028		25	100

### *In vivo* inhibition of *C. neoformans* in a mouse model by cisplatin

**Figure 1.**
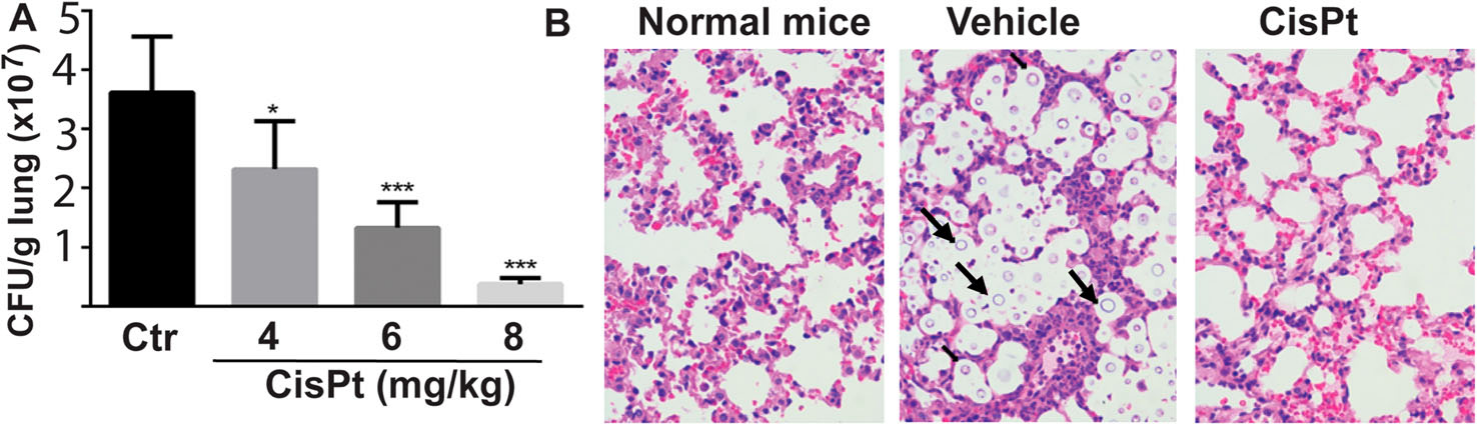
Efficacy of cisplatin in a mouse model. (A) Fungal loads of Cryptococus neoformans cells in colony forming unit (CFU) were enumerated in the lungs of mice after treatment with the vehicle control (saline) and cisplatin for 4 days. Six mice per group were used. Data are expressed as mean+SD; the experiments were performed three times. (*) p<.05, (**) p<.01, and (***) p<0.001. (B) Light micrographs of Haemotoxylin and Eosin staining sections of the lungs after treatment with cisplatin. The cryptococci are indicated by arrows and are absent in the 8 mg/kg cisplatin treatment.

On the basis of the inhibition potency of cisplatin on intein-containing cryptococcal species, we further examined its potential *in vivo* efficacy in a murine infection model of cryptococcosis. Mice were infected with *Cne* through intranasal inhalation. Cisplatin was administered by intravenous injection once daily over a 4-day dosing regimen. Fungal burden within lungs was quantified 4 days after infectious challenge. We found that the fungal burden in sham-treated mice exceeded 7 log_10_ CFU/g lungs. In contrast, in the cisplatin-treated group, the fungal burden was significantly reduced in a dose-dependent manner, with nearly a 2 log_10_ reduction of *Cne* CFU/g lungs achieved at the 8 mg/kg dose (Figure 1(A)). In contrast to the presence of abundant cryptococcal cells in the lung in sham-treated mice, no fungal cells were observed in the sections of lungs treated with cisplatin using H&E staining (Figure 1(B)).

### Development of split luciferase complementation-based Prp8 splicing assay

**Figure 2.**
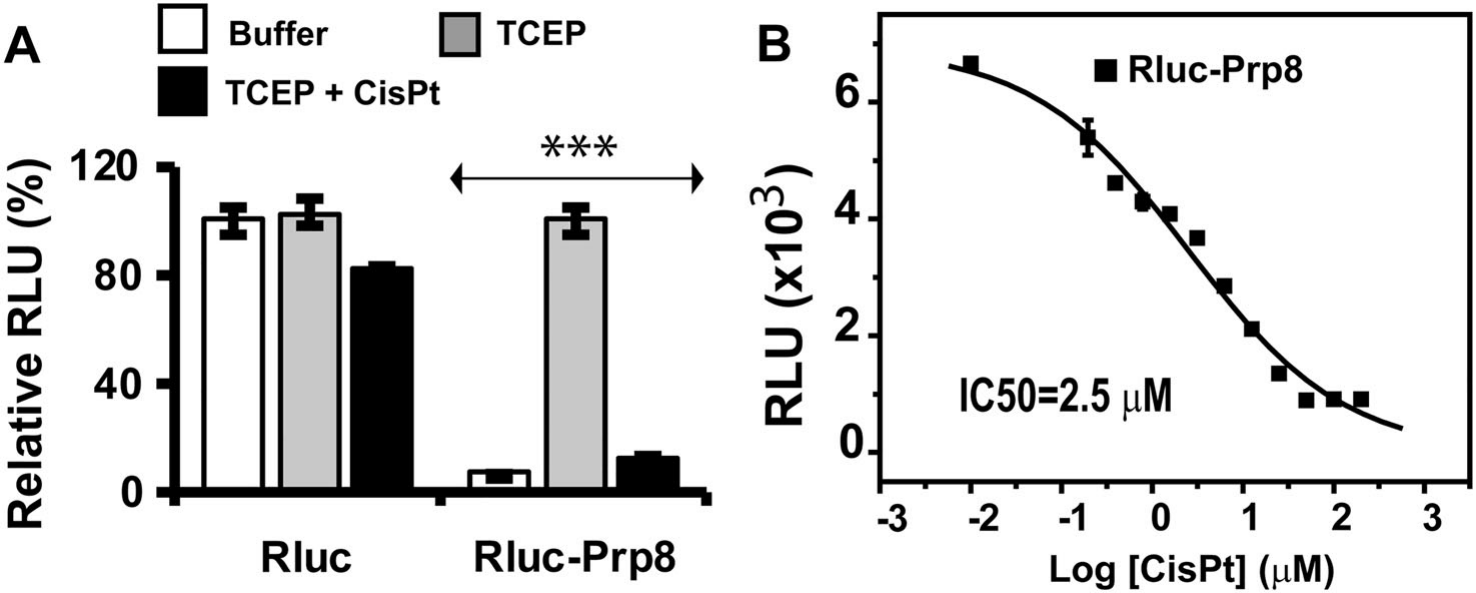
Cisplatin is a potent inhibitor of the Prp8 intein in vitro. (A) The Prp8 intein splicing assay based on split luciferase. RLuc (2 nM) or RLuc-Prp8 (2 nM) was used with DMF or cisplatin (40 µM) with/without 100 µM TCEP. Reactions were incubated at room temperature for 18 h, followed by luminescence detection. N=8. ***, p<.001. (B) Dose-response fitting of inhibition of splicing of the RLuc-Prp8 by cisplatin. RLuc-Prp8 (2 nM) was used. Cisplatin was in twofold serial dilutions with concentrations ranging from 100 µM (30 µg/ml) to 0.78 µM (0.23 µg/ml). N=3.

To investigate if the mechanism of inhibition of cryptococcal fungi by cisplatin was Prp8 intein-related, we returned to *in vitro* studies and established a Prp8 intein splicing assay based on split luciferase technology [33, 44]. To facilitate purification, we engineered a His-tag in the Prp8 intein between residues 133 and 134, a region determined to be structurally flexible and tolerant of other elements, such as a homing endonuclease domain in other inteins. Such insertions do not generally affect intein splicing activities [45, 46]. Consistently, our results verified that the His-tag inserted Prp8 intein is splicing competent (Figure 2(A)). To construct a luciferase splicing reporter, we inserted the His-tagged *Cga* Prp8 intein between the *Renilla reniformis* luciferase (RLuc) residues 229 and 230, a site commonly used in split RLuc assays [44, 47, 48]. Thus, the N-terminal (N-RLuc) and C-terminal (C-RLuc) fragments were separated by the *Cga* Prp8 intein, disrupting the RLuc integrity and leading to an inactive luciferase.

Prp8 intein splicing can reconstitute the complete RLuc, leading to a luminescence increase. To reduce Prp8 intein splicing during protein purification, we purified the split-RLuc-Prp8 at pH 9, which was previously reported to inhibit intein splicing [49]. To perform the intein splicing assay, we diluted the purified, concentrated RLuc-Prp8 into assay butter at pH 8 in a 96-well plate. As expected, the purified Prp8-RLuc did not generate luminescence when given the RLuc substrate, coelenterazine. Because the reducing reagent Tris (2-carboxyethyl) phosphine (TCEP) is known to initiate intein splicing [25, 49], we performed the same experiment in the presence of TCEP. As shown in Figure 2(A) (buffer vs. TCEP), addition of TCEP led to a significant increase in luminescence, presumably because TCEP triggers Prp8 intein splicing to generate reconstituted FL RLuc. In contrast, TCEP did not have any effect on the native RLuc luminescence (Figure 2(A)). The split RLuc assay was very robust, with as little as 2 nM fusion protein giving a strong luminescence signal upon TCEP stimulation (Figure 2(A)).

### Inhibition of Prp8 intein splicing by cisplatin

We next sought to investigate whether cisplatin can inhibit Prp8 intein splicing. As shown in Figure 2(A), cisplatin did not significantly affect the luminescence of native RLuc. In contrast, the luminescence increase of the split-RLuc-Prp8 was significantly reduced by cisplatin (Figure 2(A)). Cisplatin at 30 µM decreased TCEP-triggered RLuc luminescence eight folds. Furthermore, cisplatin inhibited Prp8 splicing in dose-dependent manner, with an *IC_50_* of 2.5 µM (Figure 2(B)). The assay *Z*’ score of 0.93 was better than the gold standard for an effective high throughput screening assay (*Z*’=0.5) [50] (Figure 2(A)).

**Figure 3.**
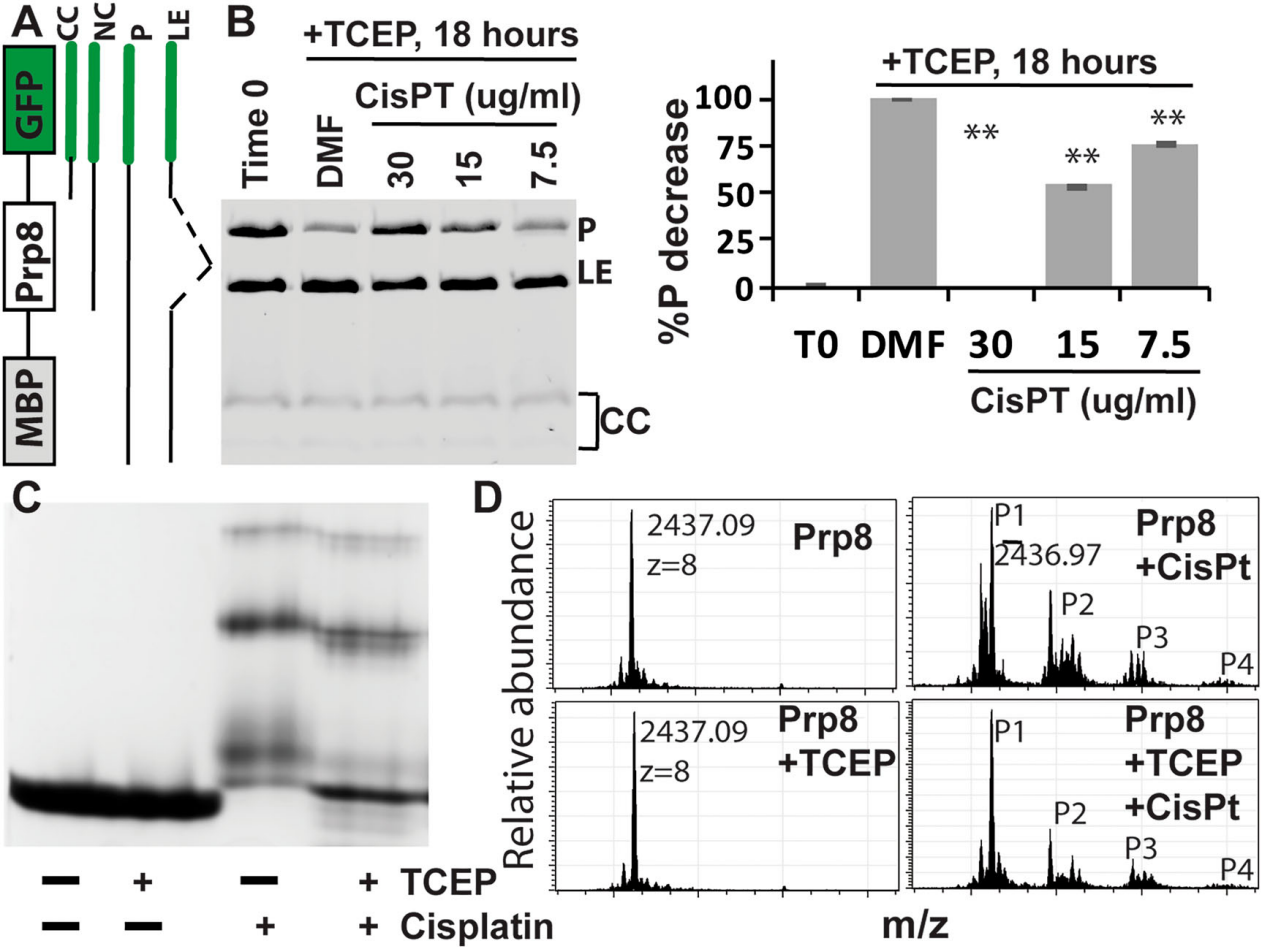
Cisplatin directly binds the Prp8 intein and inhibits Prp8 intein splicing. (A) MIG strategy and GFP-containing splicing products. (B) Dose-dependent inhibition of MIG-Prp8 splicing by CisPt with 100 µM TCEP. Left, SDS-PAGE analysis of each sample; right, relative percentage reduction of the MIG precursor upon treatment, compared to the starting material (T0). %P decrease is calculated as (%P_T0_−%P_Sample_)/(%P_T0_−%P_DMF_)*100, where %P indicates percent of the MIG precursor at time point 0 (P_T0_) and/or after ∼18 h of treatment with DMF (P_DMF_) or cisplatin at different concentrations (P_Sample_). *N*=3. **, *p*<.01. (C) Native-gel analysis of Prp8 (30 µM) in complex with CisPt (300 µM) in the presence or absence of 2 mM TCEP. (D) MS results of the WT Prp8 intein and its CisPt complex with/without TCEP.

To further investigate cisplatin inhibition of Prp8 intein splicing, we used another *in vitro* fluorescence reporter system that would allow us to observe functional Prp8 intein splicing in *E. coli* lysate. We cloned the *Cga* Prp8 intein into a reporter construct with maltose binding protein (MBP) and GFP as surrogate exteins flanking the intein (Figure 3(A)), similar to previously described assays [38]. The MBP-intein-GFP (MIG) fusion protein contains short native exteins (5 N- and C-extein amino acids) to preserve effects of extein residues at the splicing junctions. Using this system, the GFP-containing MIG precursor (P), ligated exteins product (LE), and by-products of C-terminal cleavage (CC), which often runs as multiple bands [36, 38], can be visualized in unboiled extracts by GFP fluorescence in SDS-PAGE (Figure 3(B)). At time 0, the MBP-*Cga*Prp8-GFP accumulated unspliced precursor (P) during induction, after which the precursor was autocatalytically spliced by the Prp8 intein in the presence of TCEP (DMF control in Figure 3(B)). Cisplatin treatment decreased Prp8 intein splicing in a dose-dependent manner (Figure 3(B)), indicating that cisplatin is an effective Prp8 intein splicing inhibitor and corroborating our initial *in vitro* results with split luciferase.

### Binding of cisplatin to the Prp8 intein

Previously, we showed that cisplatin binds to the active site cysteines of the mycobacterial RecA intein and induces a protein electrophoresis mobility shift in native gels only in the presence of the reducing reagent TCEP [25]. Because both the Prp8 and RecA inteins belong to the same class 1 intein family, we hypothesized that cisplatin also binds to the Prp8 intein to cause the observed inhibition. To investigate this possibility, we expressed and purified the *Cga* Prp8 intein using an intein-cleavage pXI vector [35, 36], creating a CBD-Prp8 intein fusion protein (Figure 3(C)). Incubation with dithiothreitol (DTT) stimulates N-terminal cleavage of the intein from the CBD affinity tag and generates the Prp8 intein with its native N-terminus. We performed a native gel-shift assay for cisplatin binding to the purified *Cga* Prp8 intein, in the presence and absence of TCEP. Our results show that cisplatin forms a complex with the Prp8 intein regardless of the presence or absence of TCEP, leading to the band shifts (Figure 3(C)). Binding of cisplatin to the Prp8 intein resulted in heterogeneous complexes corresponding to several bands that migrate differently in the native gel, suggesting that cisplatin binds to the Prp8 intein at multiple sites. Overall, these results indicate that the Prp8 intein does not require TCEP for binding of cisplatin, which is in contrast to the RecA intein.

To investigate the nature of cisplatin binding, we performed electrospray ionization–mass spectrometry (ESI-MS). The same purified Cga Prp8 intein was incubated overnight with or without cisplatin in the presence or absence of TCEP, and then subjected to ESI-MS analysis. Results showed that TCEP treatment did not cause any change to the mass of the *Cga* Prp8 intein (Figure 3(D)). For cisplatin-treated samples with and without TCEP, four distinct peaks were observed at identical m/z values. The data are consistent with our results from the gel shift assay, indicating that binding of cisplatin to the Prp8 intein does not require TCEP, and leads to formation of several complexes.

For the cisplatin-Prp8 intein complexes, peak 1 (P1) at m/z value of 2,436.97 corresponds to the native unmodified Prp8 intein. Three additional peaks (P2 to P4) at m/z values of 2,465.35, 2,490.22, and 2,517.22 represent the Prp8 intein in complex with one to three dissociated cisplatin molecules, respectively. Upon deconvolution, the mass differences are 227 Da between peaks P1 and P2, 426 Da between P1 and P3, and 642 Da between P1 and P4. Therefore, peak 2 accounts for the Prp8 intein in complex with a partially dissociated cisplatin, one Pt atom and two NH_2_ groups; peak 3 corresponds to the complex formed by the Prp8 intein, two completely dissociated Pt atoms, and two water molecules; and peak 4 represents the complex of one Prp8 intein, three dissociated Pt atoms and three water molecules. Our results are slightly different from the RecA intein work, in which two Pt ions were both found to bind in a completely dissociated form [25]. Although two cisplatin molecules were found in completely dissociated form in the Prp8-cisplatin complex, which is similar to that in the RecA-cisplatin complex, the third cisplatin in the Prp8-cisplatin complex was found to be partially dissociated with two NH_2_ groups retained.

### Cisplatin inhibited Prp8 intein splicing by binding to the active site of the Prp8 intein

**Figure 4.**
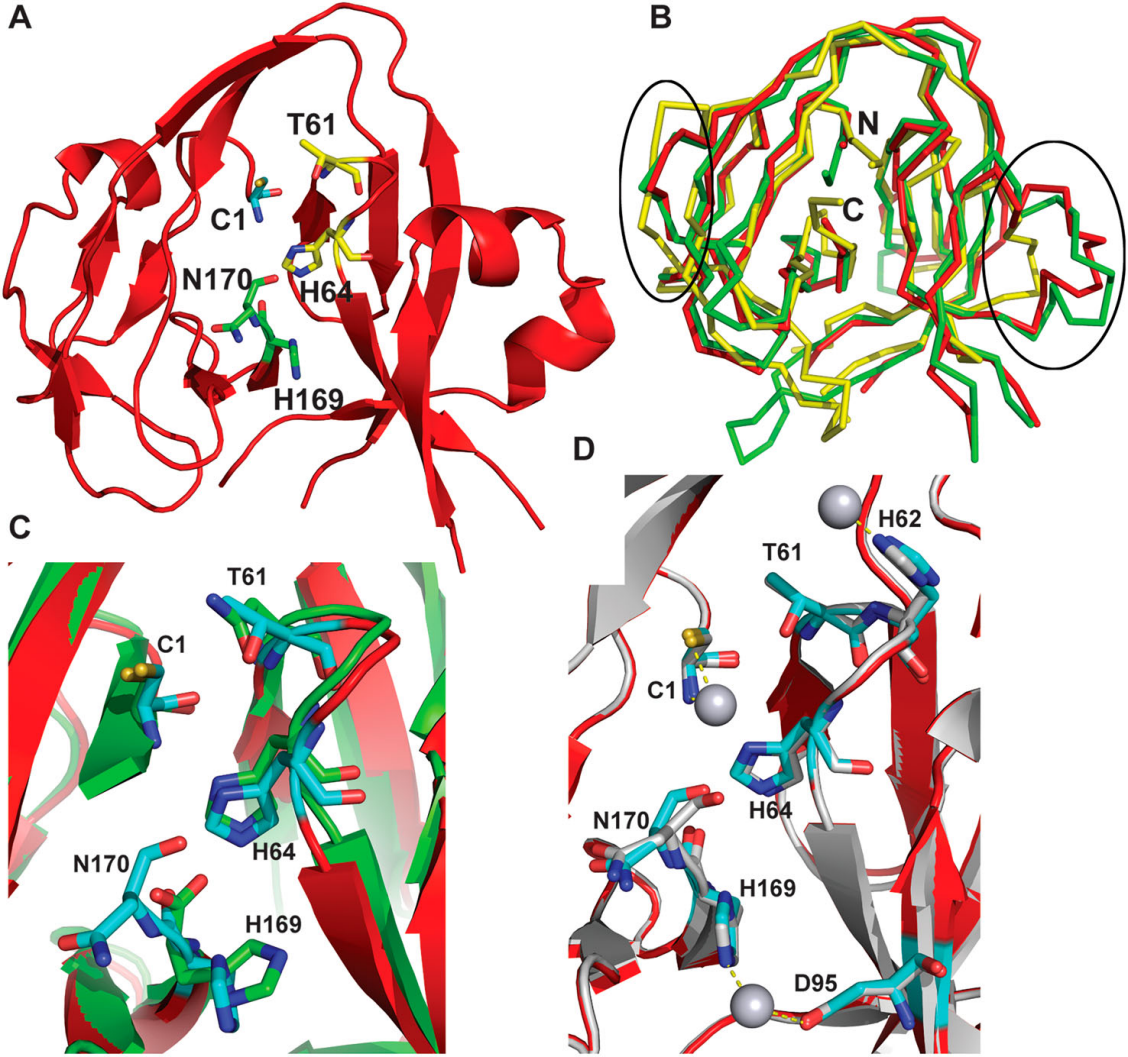
Crystal structure of the *Cga* Prp8 intein and its complex with cisplatin. (A) Overall structure of the *Cga* Prp8 intein. Active site residues in the conserved blocks are shown in stick-representation. (B) Superimposition of the structure of the *Cga* Prp8 intein with those of the *Sce* VMA1 and the *Ssp* DnaB inteins. Regions that show large structural differences between these structures are circled. N- and C-terminus are labeled. (C) Superimposition of the active site residues of the Prp8 and VMA1 inteins. (D) Structural superimposition of the native and cisplatin-bound Prp8 inteins and close-up view of interactions between the Prp8 intein and the Pt atom of cisplatin (sphere). Ligands for the platinum atom are linked with dashed lines.

To better understand the mechanism of inhibition, we determined the crystal structure of the *Cga* Prp8 intein using a pET28a construct, which is composed of a 42-residue N-terminal His-tag, the Prp8 intein (amino acids 1-170), and the Prp8 C-terminal extein residue (Ser171) (Supplemental Table 1). Crystals of the apo form of the Prp8 intein were first grown with a space group *P* 4_1_ 2_1_ 2 and three molecules per asymmetric unit. Attempts to solve the structure using the molecular replacement method with structures of various bacterial inteins as search models failed to generate a solution. We therefore generated seleno-methionine (Se-Met)-substituted Prp8 intein and determined the structure using the multiple anomalous dispersion phasing method (Supplemental Table 1). The structures of all three copies of the Prp8 intein in the asymmetric unit contain the Prp8 residues 1-72, 90-118, and 132–170 (Figure 4(A)). The structures for the loops composed of residues 73–89 and 119–131 could not be determined due to lack of electron density and these regions are presumably disordered. In the structures of other inteins, the regions similar to the Prp8 119–131 are also disordered because insertions such as endonuclease domains are frequently inserted into this region [51–53]. Although the Prp8 intein was expressed and crystallized as a His-tag fusion protein containing the catalytic centre residues Cys1 and Ser171, no electron density was observed for the long His-tag (42 residues) from the vector and the C-terminal residue, Ser171, as they likely self-spliced during the crystallization process.

The apo form of the Prp8 intein displays a canonical horseshoe-shape structure (Figure 4(A)), typical for a class 1 intein [18–21]. A structure similarity search indicated that the Prp8 intein is more similar to the VMA1 intein of *Saccharomyces cerevisiae (Sce)* (RMSD of 1.3 Å) than to bacterial inteins, such as the DnaB intein from *Synechocystis sp*. PCC 6803 (RMSD of 2.0 Å) (Figure 4(B)). The results are consistent with the notion that both VMA1 and Prp8 proteins hosting inteins are nuclear proteins from fungal species, whereas the DnaB intein is from a more distant bacterial species. The results suggest that the fungal inteins evolved from a common ancestor.

**Figure 5.**
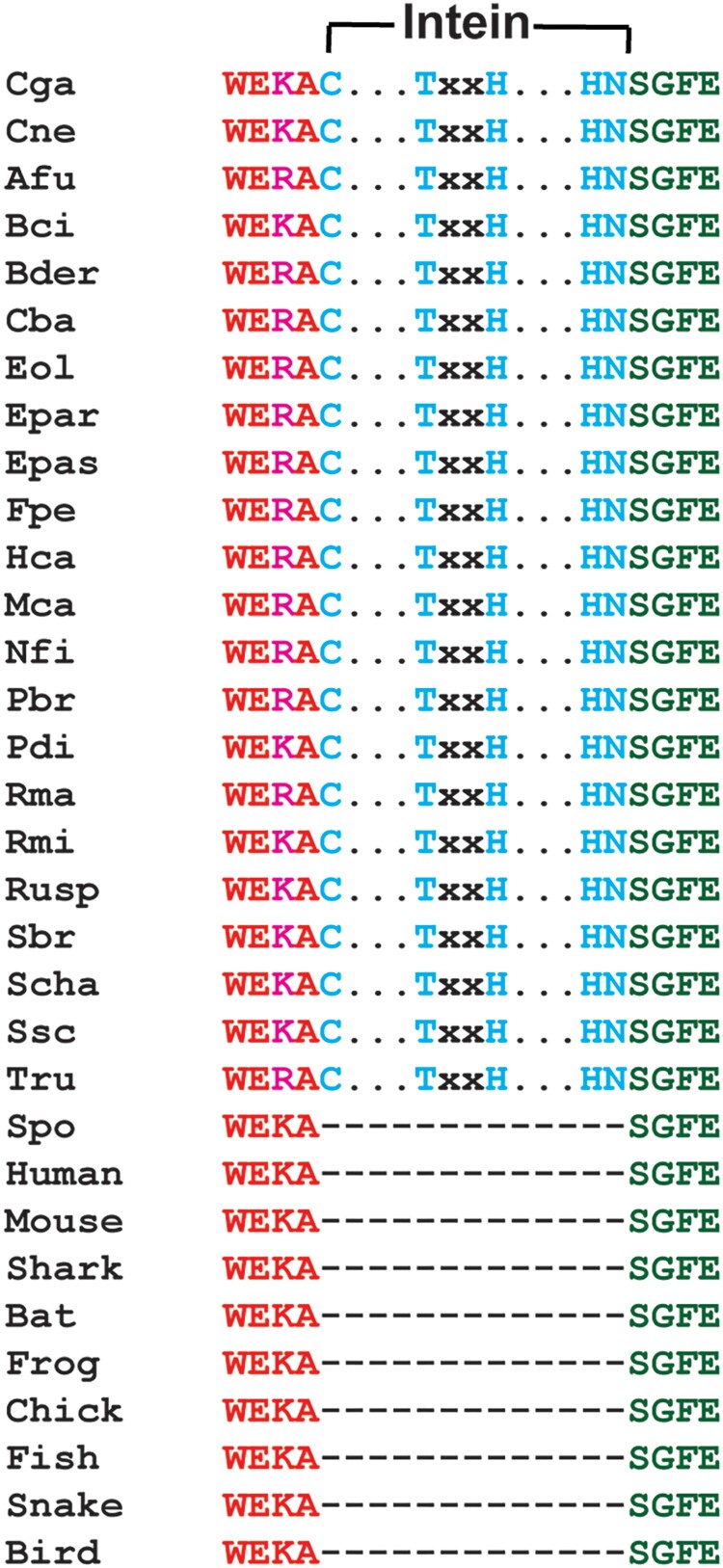
Alignment of sequences of intein-containing Prp8s of representative fungi known to cause human diseases with those of representative intein-free Prp8s at the intein insertion site. Conserved intein elements are in cyan. Abbreviations of organisms not defined in main text: *Blastomyces dermatitidis (Bder), Botrytis cinerea (Bci), Cladophialophora bantiana (Cba), Emergomyces pasteurianus (Epas), Emmonsia parva (Epar), Exophiala oligosperma (Eol), Fonsecaea pedrosoi (Fpe), Histoplasma capsulatum (Hca), Microsporum canis (Mca), Neosartorya fischeri (Nfi), Paracoccidioides brasiliensis (Pbr), Penicillium digitatum (Pdi), Rhinocladiella mackenziei (Rma), Rhizopus microsporus (Rmi), Sporothrix brasiliensis (Sbr), Sporothrix schenckii (Ssc), Stachybotrys chartarum (Scha), Trichophyton rubrum (Tru)*, and chicken (Chick).

Although the Prp8 intein has less than 30% sequence identity with other inteins, the active site residues are absolutely conserved in sequence among Prp8 intein-containing organisms, many of which are significant human pathogens (Figure 5, Supplementary Fig. 1A,B). Our results indicate that they are also conserved in structure (Figure 4(C)). The four main conserved sequence motifs (called blocks A, B, F, and G) form the protein splicing active site. As shown in Figure 4(C), the catalytic centre composed of the Prp8 residues C_1_ (block A), T_61_XXH_64_ (block B), and H_169_N_170_ (block G) is superimposed very well with that of VMA1, indicating structural conservation. These residues, although far from each other in sequence, are arranged in close proximity through the horseshoe-shape 3D-structure (Figure 4(A)). It has been shown that these residues are essential for intein splicing, involving a four-step splicing mechanism for a class 1 intein [18]. Step 1 is an amide-to-(thio)ester rearrangement of the peptide bond, *via* nucleophilic attack of the bond by the adjacent cysteine (Cys1 in Prp8). Step 2 is a transesterification reaction that transfers the N-extein from the side chain of the first intein residue to the side chain of the first C-extein residue. Step 3 is cyclization of the terminal asparagine (Asn170 in Prp8) coupled to peptide bond cleavage. Step 4 involves the spontaneous thioester-to-amide rearrangement of the bond linking the exteins to form a stable peptide bond. Overall, the structural and sequence conservations of the active site residues of the apo Prp8 intein suggests that Prp8 intein follows the class 1 intein splicing mechanism and that the Prp8 intein structure is widely conserved across intein-containing fungi.

To further establish the mechanism of inhibition of the fungal Prp8 intein by cisplatin, we determined the crystal structure of the *Cga* Prp8 intein in complex with cisplatin. Structure comparison indicated that no significant conformational change occurred upon cisplatin binding (Figure 4(D)), with an RMSD of 0.23 Å between the apo and cisplatin-bound forms of the Prp8 intein. Similar to that in the crystal structure of the RecA intein-cisplatin complex [25], the Pt(II) atoms are bound in a dissociated form (Figure 4(D)). One Pt(II) atom coordinates to a cysteine thiolate sulphur atom and the N-terminal amino nitrogen of Cys1. The second Pt(II) atom coordinates to the NE2 atom of His169 and the OD2 atom of Asp95. The third Pt(II) is liganded to the NE2 atom of His62. Among these residues, Cys1 and His169 are active site residues at the N- and C-terminal splice junction (Figure 4(D)), whereas His62 and Asp95 are not conserved in inteins.

Although cisplatin binds to the N-terminal active site Cys residues in a similar fashion in the Prp8-cisplatin and RecA-cisplatin complexes, significant differences were observed for cisplatin binding by the Prp8 and RecA inteins at the C-terminal active sites (Supplemental Fig. 2A). In the RecA-cisplatin complex, one cisplatin molecule was stabilized by Cys+1, His439, a water molecule, and a TCEP molecule at the C-terminal splicing site [25]. In contrast, cisplatin was coordinated by His169 (equivalent to His439 in RecA) and Asp95 (equivalent to Trp81 in RecA) at the Prp8 C-terminal site (Supplemental Fig. 2B). TCEP was not observed in the Prp8-cisplatin complex. The result agrees well with our biochemical data indicating that the binding of cisplatin to the Prp8 intein does not require TCEP (Figure 3(C)). Structural and sequence comparison indicated that the C-terminal active sites did not overlap very well between the two complex structures (Supplemental Fig. 2A). The RecA intein His439 is extended one register longer than the equivalent Prp8 His169. The result is that the RecA intein C-terminal site residues protrude from the molecule and leave plenty of space to accommodate a TCEP molecule to coordinate cisplatin. In contrast, cisplatin binds to the Prp8 intein C-terminal site in a much deeper position and is coordinated by a second ligand, Asp95. This position in the RecA intein is occupied by a Trp residue. The bulky Trp prevents the coordination of cisplatin to the RecA intein similarly as to the Prp8 intein. Nonetheless, cisplatin inhibits the splicing of the RecA and Prp8 inteins equivalently by binding to the active site residues.

In a mutational study of these platinum-binding residues, H62A and D95A retained splicing activity, whereas C1A and H169A mutants were inactive, as anticipated, given their catalytic roles (Supplementary Fig. 3). Overall, the structure and mutagenesis results suggest that cisplatin inhibited Prp8 intein splicing by binding to the active site residues.

### Resistance of cisplatin by overexpression of the Prp8 intein in *C. gattii*

**Figure 6.**
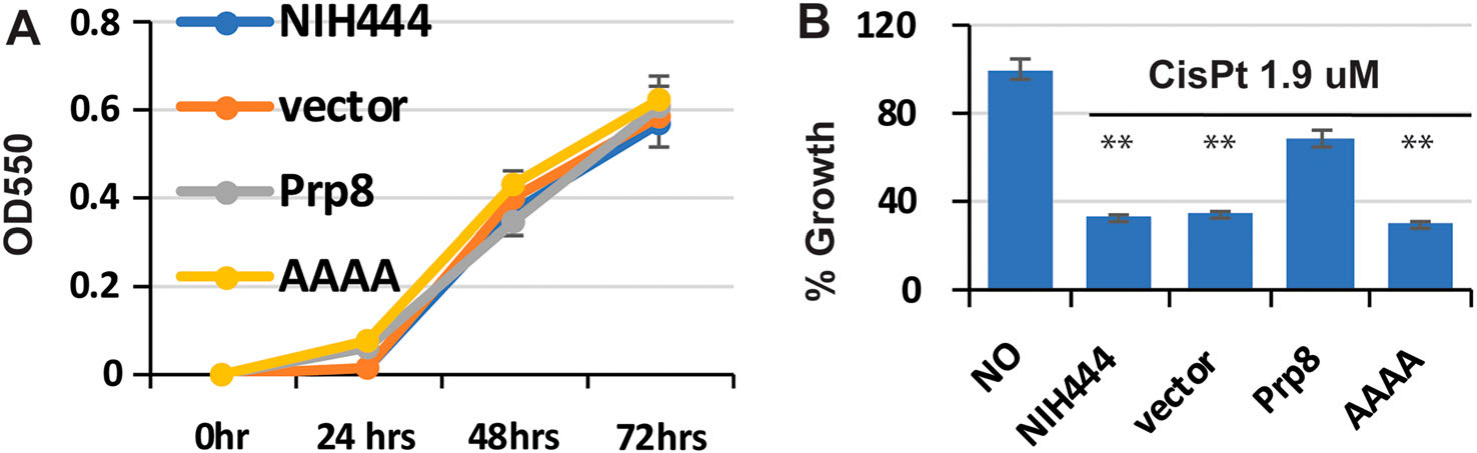
Overexpression of the Prp8 intein in the *Cga* NIH444 strain confers cisplatin resistance. (A) Transformation of NIH444 with empty vector, Prp8, and AAAA mutant does not affect the growth of NIH444. Transformed cells were cultured and monitored by OD_550_ at the time point indicated. *N*=3. (B) Transformation of the WT, but not the AAAA Prp8 mutant, into NIH444 results in resistance to cisplatin treatment. Transformed NIH444 cells were incubated with 1.9 µM cisplatin at 30°C for 48 hours. OD_550_ was measured. The OD_550_ value for untransformed NIH444 (control) in the absence of cisplatin was set as 100%. All others were expressed as a percentage of the control. *N*=3. **, *p*<.01.

To further investigate cisplatin inhibition, we performed an overexpression experiment. The *Cga* Prp8 intein-containing eight N-extein residues flanking the intein was cloned into the pXL1-PTEF1 vector under the control of the strong promoter TEF1 [54]. A non-binding AAAA mutant (C1A/H62A/D95A/H169A) was synthesized by Gene Universal as a negative control. The empty vector, WT, and the AAAA mutant plasmid were transformed into *C. gattii* NIH444. We first evaluated the growth kinetics (Figure 6(A)). Our results showed that transformation of the empty vector, Prp8 intein, and AAAA mutant did not affect the growth of *C. gattii* NIH444 in the absence of inhibitor. We next evaluated the growth inhibition of the native and transformed cells in the presence of cisplatin. As shown in Figure 6(B), cisplatin at 1.9 µM significantly reduced the growth of native NIH444. Transformation of empty vector did not alter the growth inhibition by cisplatin. In contrast, overexpression of the wild-type (WT) Prp8 intein in NIH444 conferred cisplatin resistance, whereas overexpression of the inactive AAAA Prp8 intein mutant did not lead to cisplatin resistance. Our result is consistent with drug sequestration by the Prp8 splicing domain and supports the Prp8 intein as the intracellular target of cisplatin.

## Discussion

Invasive fungal infection (IFI) treatment is usually achieved with combination therapy for a lengthy period of time, ranging from 12–14 weeks to life-long [14, 55–58]. The mainstay drugs against IFIs caused by *Cryptococcus* and *Aspergillus* fungi include: amphotericin B, flucytosine, the triazoles (such as fluconazole, voriconazole, posaconazole, itraconazole), and caspofungin (in combination therapy only) [14, 55–57]. Despite the availability of these antifungal drugs, mortality rates associated with these infections often exceed 50%. The rate and mortality of CM and aspergillosis relapses are so high that life-long therapy is required [59–61]. In addition, because the targets of these drugs are not specific for fungal cell walls or membranes, these drugs are frequently associated with severe side effects, high toxicity, and have many serious drug–drug interactions [62–64]. Moreover, because of the lengthy treatment, drug resistance has become a significant problem [15–17]. Overall, these results present an urgent and immediate need to develop new anti-IFI drugs with novel mechanisms.

Many pathogenic microbes contain a unique mobile element, called an intein, the presence of which often disrupts the normal function of the host protein. Upon intein splicing, a mature and functional host protein is generated by ligation of the N-terminal and C-terminal regions, called exteins. Due to their unique features, microbial inteins are becoming attractive drug targets [22, 24, 25, 28]. Inhibition of intein splicing could be achieved in multiple ways. Because intein splicing proceeds through four steps, inhibition of any of the four steps will lead to malfunction of the host protein. Many fungal species contain inteins in different genes [65, 66], including but not limited to *VMA, THRRS*, and *GLT1* of *Candida* species and Prp8 of *Cryptococcus, Aspergillus, Histoplasma*, and *Blastomyces* (Figure 5, Supplementary Fig. 1A,B). The presence/absence of intein in fungal species was found to be sporadic. For example, within *Candida* species, although inteins are not found in *Candida* species such as *C. albicans, C. auris, and C. boidinii*, many other *Candida* species such as *C. glabrata, C. maltosa, and C. metapsilosis* contain inteins in their genes. In fact, more than one intein was found in the genomes of some *Candida* species [66]. The presence/absence of inteins do not seem to correlate to the pathogenicity of a particular fungal species. Although it is not clear why certain fungal species do or do not contain inteins, environmental and other factors such as clonal nature and mating occurrence could contribute to this selection [66]. Nontheless, inteins have been found to play important roles in post-translational regulation of gene expression [21, 38, 67]. Inteins have also been found to be useful as molecular marker and drug target [22, 25, 28].

Many of Prp8 intein-containing fungi including *Cryptococcus, Aspergillus, Histoplasma* and *Blastomyces* are significant human pathogens [26–29]*.* The Prp8 intein belongs to the class 1 intein family [26, 28, 29]. Prp8 is also known to be essential for fungal growth*,* as a plasmid-borne copy of the gene was required for survival in a Prp8 knock-out strain [68]. The fungal Prp8 inteins have been demonstrated to be active in protein splicing [28, 29, 69]. Inactive Prp8 mutants have been observed to lead to cell death in an *in vivo* Prp8-splicing dependent growth assay [70].

In this paper, we investigated the potential of developing fungal Prp8 inteins as drug targets. We devised a highly robust Prp8 intein splicing assay based on split luciferase, showing that the split luciferase construct at as low as 2 nM concentration generated significant gain-of-luminescence upon TCEP treatment to activate splicing. These assays displayed high signal-to-background ratios and *Z*’ scores of above 0.9. Therefore, they are suitable for high throughput screenings for new inhibitors of intein splicing.

We investigated the potential of cisplatin as an inhibitor of Prp8 intein splicing. Cisplatin is known as a potent splicing inhibitor of inteins encoded by *M. tuberculosis* [24, 25]. Our results indicated that cisplatin is also a potent inhibitor of Prp8 intein splicing. The inhibition potency (IC_50_) was determined to be in the micromolar range, which is comparable to that determined for the *M. tuberculosis* inteins. However, in contrast to the *M. tuberculosis* inteins, which only bind cisplatin in the presence of TCEP, the fungal Prp8 intein bound cisplatin regardless of the presence or absence of TCEP. This conclusion was confirmed by both protein gel mobility shift assay and ESI-MS analyses. Indeed, this makes cisplatin as better inhibitor of the Prp8 intein than the RecA intein, given it does not require the external reducing agent.

We also demonstrated that cisplatin not only inhibited the Prp8 intein splicing *in vitro*, but also inhibited the growth of representative Prp8 intein-containing fungi such as different strains of *C. neoformans* and *C. gattii*. In contrast, cisplatin did not inhibit the growth of *C. albicans*, a fungus without the Prp8 intein, at comparable concentrations of cisplatin. Furthermore, cisplatin treatment significantly reduced the fungal load in a lung infection mouse model of *C. neoformans*. These data provide pharmacological demonstration that cisplatin provides a beneficial antifungal therapeutic effect in a systemic infection model of cryptococcosis and broadens the relevance of the Prp8 intein as an important drug target for developing novel antifungal agents.

Similar to other inteins, the Prp8 intein shows a canonical horseshoe-shape structure, with active-site residues highly conserved in both sequence and structure. Three cisplatin molecules were found to bind one Prp8 intein, with two bound to active-site residues, the mutation of which resulted in either total loss or great reduction of cisplatin binding. Using an overexpression strategy, we also found that overexpression of WT intein, but not a mutant defective in cisplatin binding, conferred cisplatin resistance in *C. gattii*, suggesting that cisplatin targets the Prp8 intein to inhibit fungal growth.

We sought to determine how inhibition of the Prp8 intein alters the function of host protein Prp8. The intein-free parts of the Prp8 protein are known to be well conserved with an overall 61% identity from yeast to humans in their amino acid sequences [71]. The cryo-EM structure of a spliceosome containing an intein-free Prp8 from the yeast *Schizosaccharomyces pombe (Spo)* has been determined [30, 31]. We showed that Prp8s from *Cga/Cne* (83% identity) and *Spo* share high sequence identities (54% and 66% for *Cga/Cne* to *Spo* Prp8, respectively), and that the sequences at the junction region for intein insertion are strictly conserved among various fungi including significant human pathogens. Therefore, it is reasonable to believe that the spliced (intein-free) Prp8 proteins of *Cga/Cne* have 3D structures similar to that of the *Spo* Prp8. Our structural analysis indicated that the intein insertion site was directly in the RNA binding site. Positioning of the Prp8 intein structure at the intein insertion site of the *Spo* Prp8 structure [30, 31] indicates that the intein severely overlaps with the bound U2 snRNA, U6 snRNA, and intron RNA (Supplementary Fig. 1C). All of these are essential for spliceosome function. Therefore, inhibition of the protein splicing function of the Prp8 intein will likely lead to defects in RNA splicing.

In summary, our results demonstrate that the Prp8 intein is a valid drug target, inhibition of which may lead to a novel antifungal therapy. In addition, our results show that cisplatin inhibits pathogenic fungi harbouring Prp8 inteins, both *in vitro* and *in vivo*. Cisplatin is an FDA-approved chemotherapy drug used broadly in various types of cancer treatment [72, 73]. Cisplatin is also on the World Health Organization’s list of essential medicines, which are considered the most effective and safe medicines needed in a health system. Therefore, our results suggest repurposing cisplatin and derivatives to combat infections caused by Prp8 intein-containing fungi such as *C. neoformans* and *C. gattii*.

## Supplementary Material

Supplemental Material

## References

[1] Perfect JR. Fungal diagnosis: how do we do it and can we do better? Curr Med Res Opin. 2013;29(Suppl 4):3–11. doi: 10.1185/03007995.2012.761134 23621588

[2] Brown GD, Denning DW, Gow NAR, et al. Hidden killers: human fungal infections. Sci Transl Med. 2012;4:165rv113. doi: 10.1126/scitranslmed.3004404 23253612

[3] Gullo A. Invasive fungal infections: the challenge continues. Drugs. 2009;69(Suppl 1):65–73. doi: 10.2165/11315530-000000000-00000 19877737

[4] Tuite NL, Lacey K. Overview of invasive fungal infections. Methods Mol Biol. 2013;968:1–23. doi: 10.1007/978-1-62703-257-5_1 23296882

[5] Perfect JR, Bicanic T. Cryptococcosis diagnosis and treatment: What do we know now. Fungal Genet Biol. 2015;78:49–54. doi: 10.1016/j.fgb.2014.10.003 25312862 PMC4395512

[6] Chen Y, Toffaletti DL, Tenor JL, et al. The *Cryptococcus neoformans* transcriptome at the site of human meningitis. MBio. 2014;5:e01087-13. doi: 10.1128/mBio.01087-13 24496797 PMC3950508

[7] Brouwer AE, Rajanuwong A, Chierakul W, et al. Combination antifungal therapies for HIV-associated cryptococcal meningitis: a randomised trial. Lancet. 2004;363:1764–1767. doi: 10.1016/S0140-6736(04)16301-0 15172774

[8] Husain S, Wagener MM, Singh N. *Cryptococcus neoformans* infection in organ transplant recipients: variables influencing clinical characteristics and outcome. Emerg Infect Dis. 2001;7:375–381. doi: 10.3201/eid0703.017302 11384512 PMC2631789

[9] MacDougall L, Kidd SE, Galanis E, et al. Spread of *Cryptococcus gattii* in British Columbia, Canada, and detection in the Pacific Northwest, USA. Emerg Infect Dis. 2007;13:42–50. doi: 10.3201/eid1301.060827 17370514 PMC2725832

[10] Byrnes EJ, Li W, Ren P, et al. A diverse population of *Cryptococcus gattii* molecular type VGIII in southern Californian HIV/AIDS patients. PLoS Pathog. 2011;7:e1002205. doi: 10.1371/journal.ppat.1002205 21909264 PMC3164645

[11] Byrnes EJ, Li W, Lewit Y, et al. Emergence and pathogenicity of highly virulent *Cryptococcus gattii genotypes* in the northwest United States. PLoS Pathog. 2010;6:e1000850. doi: 10.1371/journal.ppat.1000850 20421942 PMC2858702

[12] Denning DW, Pleuvry A, Cole DC. Global burden of chronic pulmonary aspergillosis complicating sarcoidosis. Eur Respir J. 2013;41:621–626. doi: 10.1183/09031936.00226911 22743676

[13] Saag MS, Graybill RJ, Larsen RA, et al. Practice guidelines for the management of cryptococcal disease. Infectious Diseases Society of America. Clin Infect Dis. 2000;30:710–718. doi: 10.1086/313757 10770733

[14] Walsh TJ, Anaissie EJ, Denning D, et al. Treatment of aspergillosis: clinical practice guidelines of the Infectious Diseases Society of America. Clin Infect Dis. 2008;46:327–360. doi: 10.1086/525258 18177225

[15] Pfaller MA. Antifungal drug resistance: mechanisms, epidemiology, and consequences for treatment. Am J Med. 2012;125:S3–S13. doi: 10.1016/j.amjmed.2011.11.001 22196207

[16] Ghannoum MA, Rice LB. Antifungal agents: mode of action, mechanisms of resistance, and correlation of these mechanisms with bacterial resistance. Clin Microbiol Rev. 1999;12:501–517. doi: 10.1128/CMR.12.4.501 10515900 PMC88922

[17] Howard SJ, Cerar D, Anderson MJ, et al. Frequency and evolution of Azole resistance in *Aspergillus fumigatus* associated with treatment failure. Emerg Infect Dis. 2009;15:1068–1076. doi: 10.3201/eid1507.090043 19624922 PMC2744247

[18] Mills KV, Johnson MA, Perler FB. Protein splicing: how inteins escape from precursor proteins. J Biol Chem. 2014;289:14498–14505. doi: 10.1074/jbc.R113.540310 24695729 PMC4031507

[19] Aranko AS, Wlodawer A, Iwai H. Nature's recipe for splitting inteins. Protein Eng Des Sel. 2014;27:263–271. doi: 10.1093/protein/gzu028 25096198 PMC4133565

[20] Eryilmaz E, Shah NH, Muir TW, et al. Structural and dynamical features of inteins and implications on protein splicing. J Biol Chem. 2014;289:14506–14511. doi: 10.1074/jbc.R113.540302 24695731 PMC4031508

[21] Novikova O, Topilina N, Belfort M. Enigmatic distribution, evolution, and function of inteins. J Biol Chem. 2014;289:14490–14497. doi: 10.1074/jbc.R114.548255 24695741 PMC4031506

[22] Paulus H. Inteins as targets for potential antimycobacterial drugs. Front Biosci. 2003;8:s1157–s1165. doi: 10.2741/1195 12957838

[23] Paulus H. Protein splicing inhibitors as a new class of antimycobacterial agents. Drugs Future. 2007;32:973–984. doi: 10.1358/dof.2007.032.11.1140690

[24] Zhang L, Zheng Y, Callahan B, et al. Cisplatin inhibits protein splicing, suggesting inteins as therapeutic targets in mycobacteria. J Biol Chem. 2011;286:1277–1282. doi: 10.1074/jbc.M110.171124 21059649 PMC3020735

[25] Chan H, Pearson CS, Green CM, et al. Exploring intein inhibition by platinum compounds as an antimicrobial strategy. J Biol Chem. 2016;291:22661–22670. doi: 10.1074/jbc.M116.747824 27609519 PMC5077202

[26] Butler MI, Goodwin TJ, Poulter RT. A nuclear-encoded intein in the fungal pathogen *Cryptococcus neoformans*. Yeast. 2001;18:1365–1370. doi: 10.1002/yea.781 11746598

[27] Butler MI, Poulter RT. The PRP8 inteins in *Cryptococcus* are a source of phylogenetic and epidemiological information. Fungal Genet Biol. 2005;42:452–463. doi: 10.1016/j.fgb.2005.01.011 15809009

[28] Liu XQ, Yang J. Prp8 intein in fungal pathogens: target for potential antifungal drugs. FEBS Lett. 2004;572:46–50. doi: 10.1016/j.febslet.2004.07.016 15304322

[29] Pearl EJ, Tyndall JD, Poulter RT, et al. Sequence requirements for splicing by the Cne PRP8 intein. FEBS Lett. 2007;581:3000–3004. doi: 10.1016/j.febslet.2007.05.060 17544410

[30] Hang J, Wan R, Yan C, et al. Structural basis of pre-mRNA splicing. Science. 2015;349:1191–1198. doi: 10.1126/science.aac8159 26292705

[31] Yan C, Hang J, Wan R, et al. Structure of a yeast spliceosome at 3.6-angstrom resolution. Science. 2015;349:1182–1191. doi: 10.1126/science.aac7629 26292707

[32] Brecher M, Li Z, Liu B, et al. A conformational switch high-throughput screening assay and allosteric inhibition of the flavivirus NS2B-NS3 protease. PLoS Pathog. 2017;13:e1006411. doi: 10.1371/journal.ppat.1006411 28542603 PMC5462475

[33] Li Z, Brecher M, Deng Y-Q, et al. Existing drugs as broad-spectrum and potent inhibitors for Zika virus by targeting NS2B-NS3 interaction. Cell Res. 2017;27:1046–1064. doi: 10.1038/cr.2017.88 28685770 PMC5539352

[34] Higuchi R, Krummel B, Saiki RK. A general method of in vitro preparation and specific mutagenesis of DNA fragments: study of protein and DNA interactions. Nucleic Acids Res. 1988;16:7351–7367. doi: 10.1093/nar/16.15.7351 3045756 PMC338413

[35] Hiraga K, Derbyshire V, Dansereau JT, et al. Minimization and stabilization of the *Mycobacterium tuberculosis* recA intein. J Mol Biol. 2005;354:916–926. doi: 10.1016/j.jmb.2005.09.088 16288917

[36] Kelley DS, Lennon CW, Li Z, et al. Mycobacterial DnaB helicase intein as oxidative stress sensor. Nat Commun. 2018;9:4363. doi: 10.1038/s41467-018-06554-x 30341292 PMC6195587

[37] Zhao Y, Li Z, Drozd SJ, et al. Crystal structure of mycoplasma arthritidis mitogen complexed with HLA-DR1 reveals a novel superantigen fold and a dimerized superantigen-MHC complex. Structure. 2004;12:277–288. 14962388 10.1016/j.str.2004.01.008 PMC3923524

[38] Topilina NI, Green CM, Jayachandran P, et al. Sufb intein of *Mycobacterium tuberculosis* as a sensor for oxidative and nitrosative stresses. Proc Natl Acad Sci U S A. 2015;112:10348–10353. doi: 10.1073/pnas.1512777112 26240361 PMC4547236

[39] Otwinowski Z, Minor W. Processing of X-ray diffraction data collected in oscillation mode. Methods Enzymol. 1997;276:307–326. doi: 10.1016/S0076-6879(97)76066-X 27754618

[40] Adams PD, Afonine PV, Bunkóczi G, et al. PHENIX: a comprehensive Python-based system for macromolecular structure solution. Acta Crystallogr D Biol Crystallogr. 2010;66:213–221. doi: 10.1107/S0907444909052925 20124702 PMC2815670

[41] Emsley P, Lohkamp B, Scott WG, et al. Features and development of Coot. Acta Crystallogr D Biol Crystallogr. 2010;66:486–501. doi: 10.1107/S0907444910007493 20383002 PMC2852313

[42] Klepser ME, Ernst EJ, Ernst ME, et al. Evaluation of endpoints for antifungal susceptibility determinations with LY303366. Antimicrob Agents Chemother. 1998;42:1387–1391. doi: 10.1128/AAC.42.6.1387 9624481 PMC105609

[43] Moussa NM, Ghannoum MA, Whittaker PA, et al. Effects of cisplatin and two novel palladium complexes on *Candida albicans*. Microbios. 1990;62:165–178. 2195302

[44] Paulmurugan R, Gambhir SS. Monitoring protein-protein interactions using split synthetic renilla luciferase protein-fragment-assisted complementation. Anal Chem. 2003;75:1584–1589. doi: 10.1021/ac020731c 12705589 PMC4154785

[45] Chong S, Xu MQ. Protein splicing of the Saccharomyces cerevisiae VMA intein without the endonuclease motifs. J Biol Chem. 1997;272:15587–15590. doi: 10.1074/jbc.272.25.15587 9188443

[46] Derbyshire V, Wood DW, Wu W, et al. Genetic definition of a protein-splicing domain: functional mini-inteins support structure predictions and a model for intein evolution. Proc Natl Acad Sci U S A. 1997;94:11466–11471. doi: 10.1073/pnas.94.21.11466 9326633 PMC23508

[47] Azad T, Tashakor A, Hosseinkhani S. Split-luciferase complementary assay: applications, recent developments, and future perspectives. Anal Bioanal Chem. 2014;406:5541–5560. doi: 10.1007/s00216-014-7980-8 25002334

[48] Jiang Y, Bernard D, Yu Y, et al. Split Renilla luciferase protein fragment-assisted complementation (SRL-PFAC) to characterize Hsp90-Cdc37 complex and identify critical residues in protein/protein interactions. J Biol Chem. 2010;285:21023–21036. doi: 10.1074/jbc.M110.103390 20413594 PMC2898314

[49] Gangopadhyay JP, Jiang SQ, Paulus H. An in vitro screening system for protein splicing inhibitors based on green fluorescent protein as an indicator. Anal Chem. 2003;75:2456–2462. doi: 10.1021/ac020756b 12918990

[50] Iversen PW, Beck B, Chen YF, et al. HTS Assay Validation. doi:NBK83783 [bookaccession] (2004).

[51] Van Roey P, Pereira B, Li Z, et al. Crystallographic and mutational studies of *Mycobacterium tuberculosis* recA mini-inteins suggest a pivotal role for a highly conserved aspartate residue. J Mol Biol. 2007;367:162–173. doi: 10.1016/j.jmb.2006.12.050 17254599 PMC1852430

[52] Ding Y, Xu M-Q, Ghosh I, et al. Crystal structure of a mini-intein reveals a conserved catalytic module involved in side chain cyclization of asparagine during protein splicing. J Biol Chem. 2003;278:39133–39142. doi: 10.1074/jbc.M306197200 12878593

[53] Werner E, Wende W, Pingoud A, et al. High resolution crystal structure of domain I of the Saccharomyces cerevisiae homing endonuclease PI-SceI. Nucleic Acids Res. 2002;30:3962–3971. doi: 10.1093/nar/gkf523 12235380 PMC137108

[54] Zhao Y, Upadhyay S, Lin X. PAS domain protein Pas3 interacts with the chromatin modifier Bre1 in regulating cryptococcal morphogenesis. MBio. 2018;9:e02135-18. doi: 10.1128/mBio.02135-18 30425151 PMC6234864

[55] Perfect JR, Dismukes W, Dromer F, et al. Clinical practice guidelines for the management of cryptococcal disease: 2010 update by the infectious diseases society of america. Clin Infect Dis. 2010;50:291–322. doi: 10.1086/649858 20047480 PMC5826644

[56] Rajasingham R, Rolfes MA, Birkenkamp KE, et al. Cryptococcal meningitis treatment strategies in resource-limited settings: a cost-effectiveness analysis. PLoS Med. 2012;9:e1001316. doi: 10.1371/journal.pmed.1001316 23055838 PMC3463510

[57] Kontoyiannis DP, Lionakis M, Lewis R, et al. Zygomycosis in a tertiary-care cancer center in the era of *Aspergillus*-active antifungal therapy: a case-control observational study of 27 recent cases. J Infect Dis. 2005;191:1350–1360. doi: 10.1086/428780 15776383

[58] Denning DW, Park S, Lass-Florl C, et al. High-frequency triazole resistance found In nonculturable *Aspergillus fumigatus* from lungs of patients with chronic fungal disease. Clin Infect Dis. 2011;52:1123–1129. doi: 10.1093/cid/cir179 21467016 PMC3106268

[59] Zuger A, Louie E, Holzman RS, et al. Cryptococcal disease in patients with the acquired immunodeficiency syndrome. Diagnostic features and outcome of treatment. Ann Intern Med. 1986;104:234–240. doi: 10.7326/0003-4819-104-2-234 3946951

[60] Sipsas NV, Kontoyiannis DP. Clinical issues regarding relapsing aspergillosis and the efficacy of secondary antifungal prophylaxis in patients with hematological malignancies. Clin Infect Dis. 2006;42:1584–1591. doi: 10.1086/503844 16652316

[61] Boelaert JR, Goddeeris KH, Vanopdenbosch LJ, et al. Relapsing meningitis caused by persistent cryptococcal antigens and immune reconstitution after the initiation of highly active antiretroviral therapy. AIDS. 2004;18:1223–1224. doi: 10.1097/00002030-200405210-00023 15166545

[62] Gubbins PO, McConnell SA, Penzak SR. Drug interactions in infectious Diseases. New York: Humana Press; 2001.

[63] Moen MD, Lyseng-Williamson KA, Scott LJ. Liposomal amphotericin B: a review of its use as empirical therapy in febrile neutropenia and in the treatment of invasive fungal infections. Drugs. 2009;69:361–392. doi: 10.2165/00003495-200969030-00010 19275278

[64] Kauffman CA. Fungal infections. Proc Am Thorac Soc. 2006;3:35–40. doi: 10.1513/pats.200510-110JH 16493149

[65] Green CM, Novikova O, Belfort M. The dynamic intein landscape of eukaryotes. Mob DNA. 2018;9(4):1–16. 29416568 10.1186/s13100-018-0111-x PMC5784728

[66] Fernandes JA, Prandini THR, Castro MA, et al. Evolution and application of inteins in *Candida* species: a review. Front Microbiol. 2016;7:1585. doi: 10.3389/fmicb.2016.01585 27777569 PMC5056185

[67] Lennon CW, Stanger M, Belfort M. Protein splicing of a recombinase intein induced by ssDNA and DNA damage. Genes Dev. 2016;30:2663–2668. doi: 10.1101/gad.289280.116 28031248 PMC5238726

[68] Kuhn AN, Reichl EM, Brow DA. Distinct domains of splicing factor Prp8 mediate different aspects of spliceosome activation. Proc Natl Acad Sci U S A. 2002;99:9145–9149. doi: 10.1073/pnas.102304299 12087126 PMC123108

[69] Green CM, Li Z, Novikova O, et al. Spliceosomal Prp8 intein at the crossroads of protein and RNA splicing. Under review (2019). doi:10.1101/502781 PMC6805012 31600193

[70] Pearl EJ, Bokor AA, Butler MI, et al. Preceding hydrophobic and beta-branched amino acids attenuate splicing by the CnePRP8 intein. Biochim Biophys Acta. 2007;1774:995–1001. doi: 10.1016/j.bbapap.2007.05.015 17604706

[71] Grainger RJ, Beggs JD. Prp8 protein: at the heart of the spliceosome. RNA. 2005;11:533–557. doi: 10.1261/rna.2220705 15840809 PMC1370742

[72] Dasari S, Tchounwou PB. Cisplatin in cancer therapy: molecular mechanisms of action. Eur J Pharmacol. 2014;740:364–378. doi: 10.1016/j.ejphar.2014.07.025 25058905 PMC4146684

[73] Kelland L. The resurgence of platinum-based cancer chemotherapy. Nat Rev Cancer. 2007;7:573–584. doi: 10.1038/nrc2167 17625587

