# Supplementary material for: Cisplatin protects mice from challenge of Cryptococcus neoformans by targeting the Prp8 intein
Source: Emerg Microbes Infect. 2019 Jun 21;8(1):895–908. doi: 10.1080/22221751.2019.1625727 (PMC6598491; doi:10.1080/22221751.2019.1625727)
Supplement: Supplemental Material [file TEMI_A_1625727_SM4826.docx]

**Supplemental Text and Figures:**

**Title: Cisplatin protects mice from challenge of *Cryptococcus neoformans* by targeting the Prp8 intein**

Zhong Li^1,₸^, Bin Fu^2,₸^, Cathleen M. Green^3^, Binbin Liu^1,±^, Jing Zhang^1^, Yuekun Lang^1^, Sudha Chaturvedi^1,4^, Marlene Belfort^3,4^, Guojian Liao^2^* Hongmin Li^1,4*^

^1^ Wadsworth Center, New York State Department of Health, 120 New Scotland Ave, Albany, New York 12208, USA

^2^ College of Pharmaceutical Sciences, Southwest University, Chongqing 400715, China

^3^ Department of Biological Sciences and RNA Institute, University at Albany, 1400 Washington Avenue, Albany, New York 12222, USA

^4^ Department of Biomedical Sciences, School of Public Health, University at Albany, Empire State Plaza, PO Box 509, Albany, New York 12201-0509, USA

^₸^These authors contributed equally.

^±^Current address: Department of Food Science, College of Food Science and Technology, Guangdong Ocean University, Zhanjiang, Guangdong 524000, China

^*^Corresponding authors: Guojian Liao, Phone: +86-23-6825-1225, Email: [gjliao@swu.edu.cn](mailto:gjliao@swu.edu.cn); Hongmin Li, Phone: 518-473-4201, Email: [Hongmin.li@health.ny.gov](mailto:Hongmin.li@health.ny.gov)

**Supplemental Text and Figures**

**Fig. S1.** Inhibition of the Prp8 intein splicing could lead to disruption of interactions between the Prp8 protein and other spliceosome components.

**Fig. S2.** Sequence and structure comparison between the RecA-cisplatin (green, PDB: 5I0A) and Prp8-cisplatin (cyan) complexes.

**Fig. S3.** Impact of cisplatin-binding residues on cisplatin binding and the Prp8 intein splicing activities.

**Table S1**. Data collection and refinement statistics

**Table S2**. Primers used in the manuscript

**Validation report** for the crystal structure of the Prp8 intein of *C. gattii*, 6MWY

**Validation report** for the crystal structure of the Prp8 intein of *C. gattii* in complex with cisplatin, 6MYL


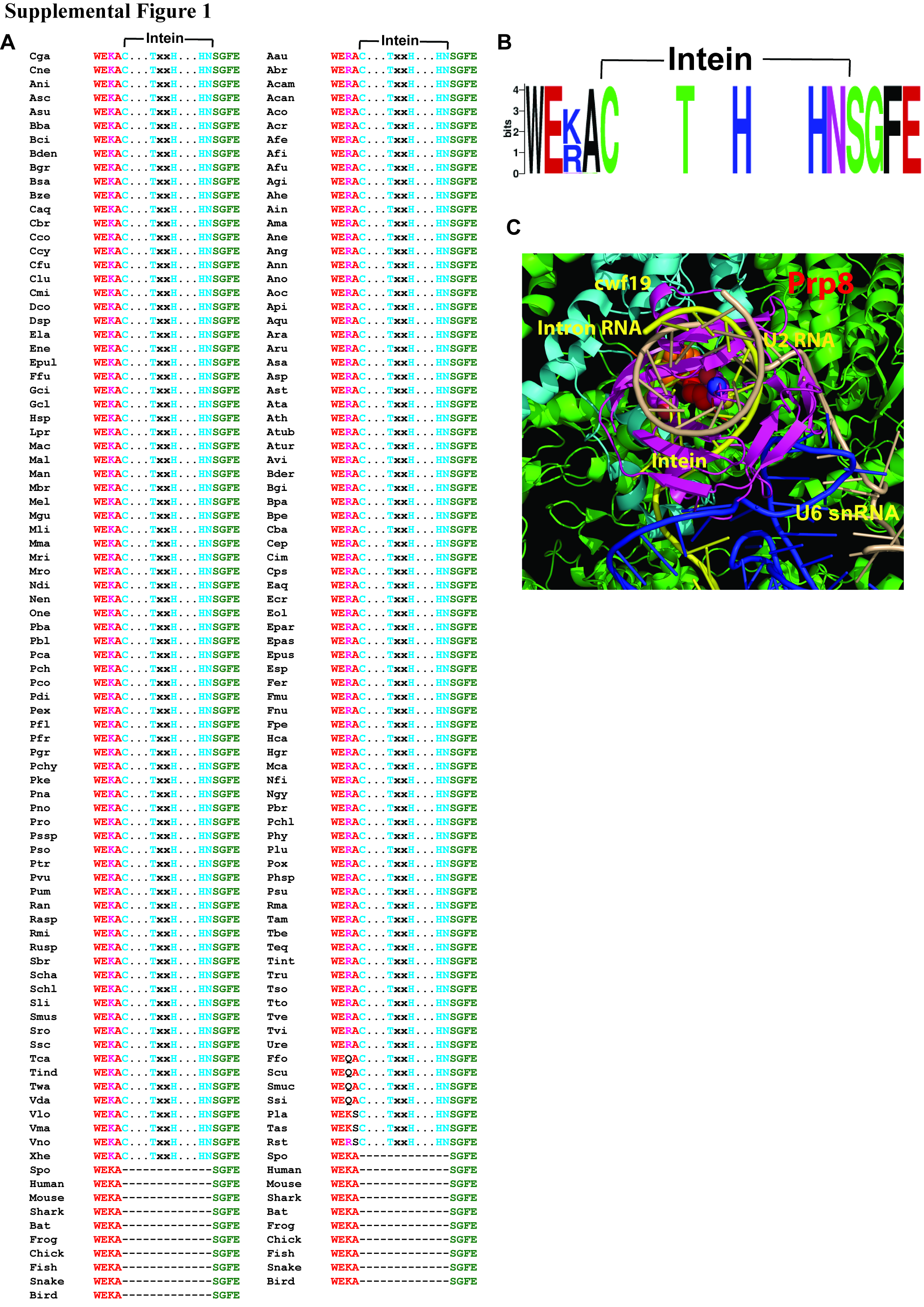


**Supplemental Figure 1.** Inhibition of the Prp8 intein splicing could lead to disruption of interactions between the Prp8 protein and other spliceosome components. **(A)** Complete sequence alignment of the representative intein-containing and intein-free Prp8s at the intein insertion site. Conserved intein elements are in cyan. Organism abbreviations: *Acytostelium subglobosum (Asu), Allomyces macrogynus (Ama), Aspergillus auratus (Aau), Aspergillus brevipes (Abr), Aspergillus campestris (Acam), Aspergillus candidus (Acan), Aspergillus costaricaensis (Aco), Aspergillus cristatus (Acr), Aspergillus fennelliae (Afe), Aspergillus fischeri (Afi), Aspergillus giganteus (Agi), Aspergillus heteromorphus (Ahe), Aspergillus indologenus (Ain), Aspergillus neoglaber (Ang), Aspergillus neoellipticus (Ane), Aspergillus neoniger (Ann), Aspergillus nidulans (Ani), Aspergillus novofumigatus (Ano), Aspergillus ochraceoroseus (Aoc), Aspergillus piperis (Api), Aspergillus quadricinctus (Aqu), Aspergillus rambellii (Ara), Aspergillus ruber (Aru), Aspergillus saccharolyticus (Asa), Aspergillus sclerotialis (Asc), Aspergillus spinosus (Asp), Aspergillus steynii (Ast), Aspergillus taichungensis (Ata), Aspergillus thermomutatus (Ath), Aspergillus tubingensis (Atub), Aspergillus turcosus (Atur), Aspergillus viridinutans (Avi), Batrachochytrium dendrobatidis (Bden), Batrachochytrium salamandrivorans (Bsa), Beauveria bassiana (Bba), Bipolaris zeicola (Bze), Blastomyces dermatitidis (Bder), Blastomyces gilchristii (Bgi), Blastomyces parvus (Bpa), Blastomyces percursus (Bpe), Blumeria graminis (Bgr), Botrytis cinerea (Bci), Capronia epimyces (Cep), Cladophialophora bantiana (Cba), Cladophialophora immunda (Cim), Cladophialophora psammophila (Cps), Clohesyomyces aquaticus (Caq), Coleophoma cylindrospora (Ccy), Coniella lustricola (Clu), Cordyceps brongniartii (Cbr), Cordyceps confragosa (Cco), Cordyceps fumosorosea (Cfu), Cordyceps militaris (Cmi), Daldinia sp (Dsp), Drechmeria coniospora (Dco), Emergomyces pasteurianus (Epas), Emmonsia crescens (Ecr), Emmonsia parva (Epar), Emmonsia sp (Esp),* *Endocarpon pusillum (Epus), Erysiphe necator (Ene), Erysiphe pulchra (Epul), Eutypa lata UCREL1 (Ela), Exophiala aquamarina (Eaq), Exophiala oligosperma (Eol), Fonsecaea erecta (Fer), Fonsecaea multimorphosa (Fmu), Fonsecaea nubica (Fnu),* *Fonsecaea pedrosoi (Fpe), Furculomyces boomerangus (Fbo), Fusarium fujikuroi (Ffu), Golovinomyces cichoracearum (Gci), Grosmannia clavigera (Gcl), Helicocarpus griseus (Hgr), Histoplasma capsulatum (Hca), Hypoxylon sp. (Hsp), Lomentospora prolificans (Lpr), Metarhizium acridum (Mac), Metarhizium album (Mal), Metarhizium anisopliae (Man), Metarhizium brunneum (Mbr), Metarhizium guizhouense (Mgu), Metarhizium majus (Mma), Metarhizium rileyi (Mri), Metarhizium robertsii (Mro), Microsporum canis (Mca), Moelleriella libera (Mli), Mortierella elongate (Mel),* *Naematelia encephala (Nen), Nannizzia gypsea (Ngy), Neonectria ditissima (Ndi), Neosartorya fischeri (Nfi), Oidium neolycopersici (One), Papiliotrema laurentii (Pla), Paracoccidioides brasiliensis (Pbr), Paracoccidioides lutzii (Plu), Penicillium baarnense (Pba), Penicillium camemberti (Pca), Penicillium chrysogenum (Pch), Penicillium coprophilum (Pco), Penicillium digitatum (Pdi), Penicillium expansum (Pex), Penicillium flavigenum (Pfl), Penicillium freii (Pfr), Penicillium griseofulvum (pgr), Penicillium italicum (Pit), Penicillium kewense (Pke), Penicillium nalgiovense (Pna), Penicillium nordicum (Pno), Penicillium oxalicum (Pox), Penicillium roqueforti (Pro), Penicillium solitum (Pso), Penicillium subrubescens (Psu), Penicillium vulpinum (Pvu), Phaeomoniella chlamydospora (Pchl), Phialophora cf. hyaline (Pchy), Phialosimplex sp (Phsp), Phycomyces blakesleeanus (Pbl), Polytolypa hystricis (Phy), Porphyra umbilicalis (Pum), Pseudogymnoascus sp. (Pssp), Pyrenophora tritici-repentis (Ptr), Rachicladosporium antarcticum (Ran), Rachicladosporium sp. (Rasp), Rhinocladiella mackenziei (Rma), Rhizopus microsporus (Rmi), Rhizopus stolonifer (Rst), Rutstroemia sp (Rusp), Salpingoeca rosetta (Sro), Smittium culicis (Scu), Smittium mucronatum (Smuc), Smittium simulii (Ssi), Sphaerulina musiva (Smus), Sporothrix brasiliensis (Sbr), Sporothrix schenckii (Ssc), Stachybotrys chartarum (Scha), Stachybotrys chlorohalonata (Schl), Scytalidium lignicola (Sli), Talaromyces amestolkiae (Tam), Tilletia indica (Tind), Tilletia walker (Twa), Tolypocladium capitatum (Tca), Trichosporon asahii (Tas), Trichophyton benhamiae (Tbe), Trichophyton equinum (Teq), Trichophyton interdigitale (Tint), Trichophyton rubrum (Tru), Trichophyton soudanense (Tso), Trichophyton tonsurans (Tto), Trichophyton verrucosum (Tve), Trichophyton violaceum (Tvi), Uncinocarpus reesii (Ure), Valsa mali (Vma), Verticillium dahliae (Vda), Verticillium longisporum (Vlo), Verticillium nonalfalfae (Vno), Xylona heveae (Xhe).* **(B)** Logo view of sequence conservation. **(C)** Modeling of the Prp8-intein (magenta) onto the *Spo* Prp8 of the spliceosome (PDB: 3JB9)**.** The Prp8 and cwf19 proteins of the spliceosome are colored green and cyan, respectively. Colors of bound RNA are: intron RNA (yellow); U2 RNA (wheat); U6 snRNA (blue). The Prp8 intein is placed with its N- and C-termini (red and blue sphere) close to the *Spo* Prp8 AS residues at the intein insertion site (red and blue sphere).

**Supplemental Figure 2**

**
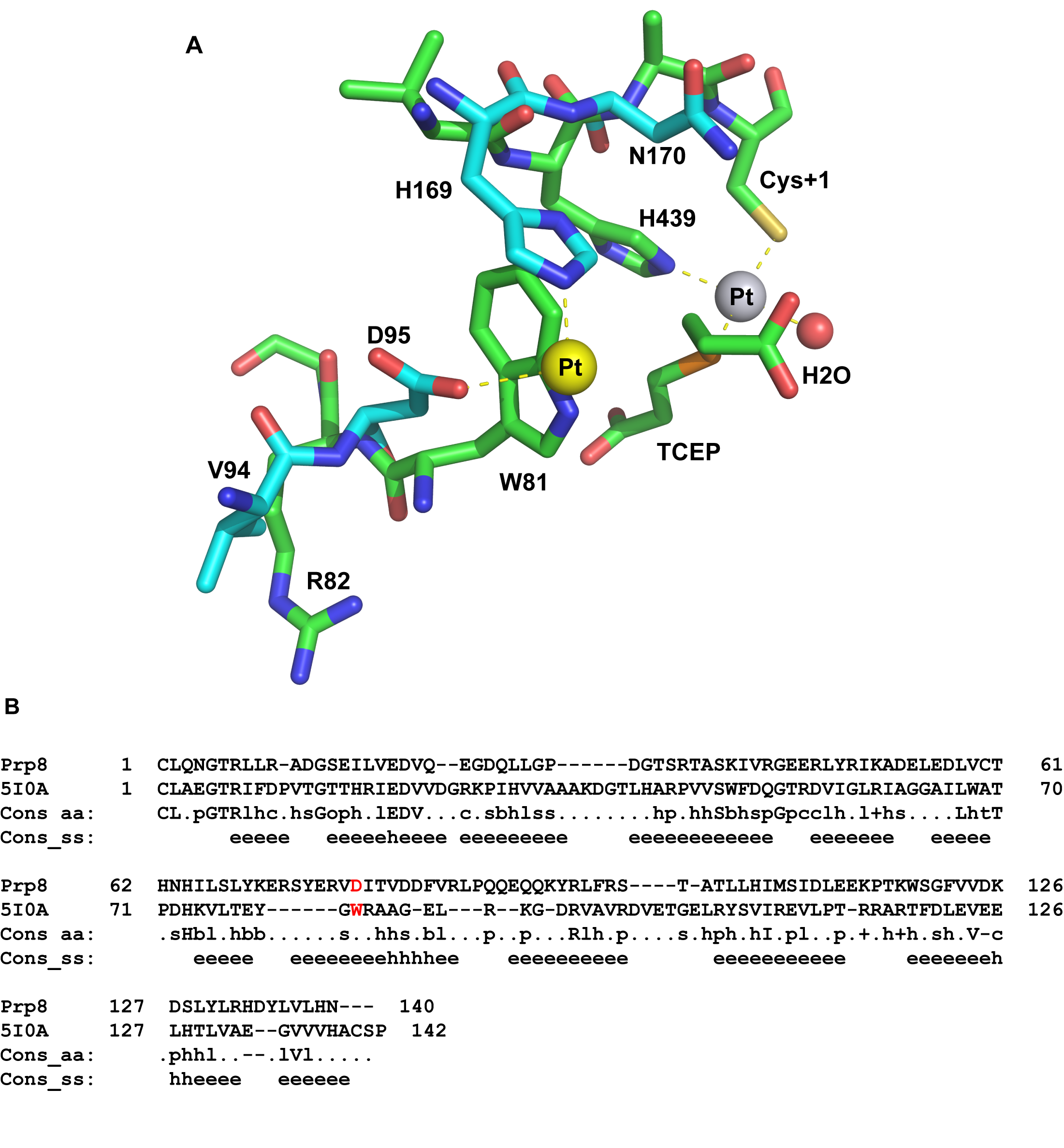
**

**Supplemental Figure 2.** Sequence and structure comparison between the RecA-cisplatin (green, PDB: 5I0A) and Prp8-cisplatin (cyan) complexes. (A). Structural comparison at the C-terminal cisplatin-binding sites between the RecA-cisplatin (green) and Prp8-cisplatin (cyan) complexes. Atomic colors as follows: oxygen, red; carbon, cyan (Prp8) and green (RecA); nitrogen, blue. Pt (yellow for Prp8 and grey for RecA) and water (red) were in sphere representation. (B). Structure-based sequence alignment using the PROMALS3D multiple sequence and structure alignment server (<http://prodata.swmed.edu/promals3d/promals3d.php> ). D95 and W81 were colored red. Consensus amino acid (cons_aa) and consensus secondary structure (cons_ss) were shown under the sequences.

**
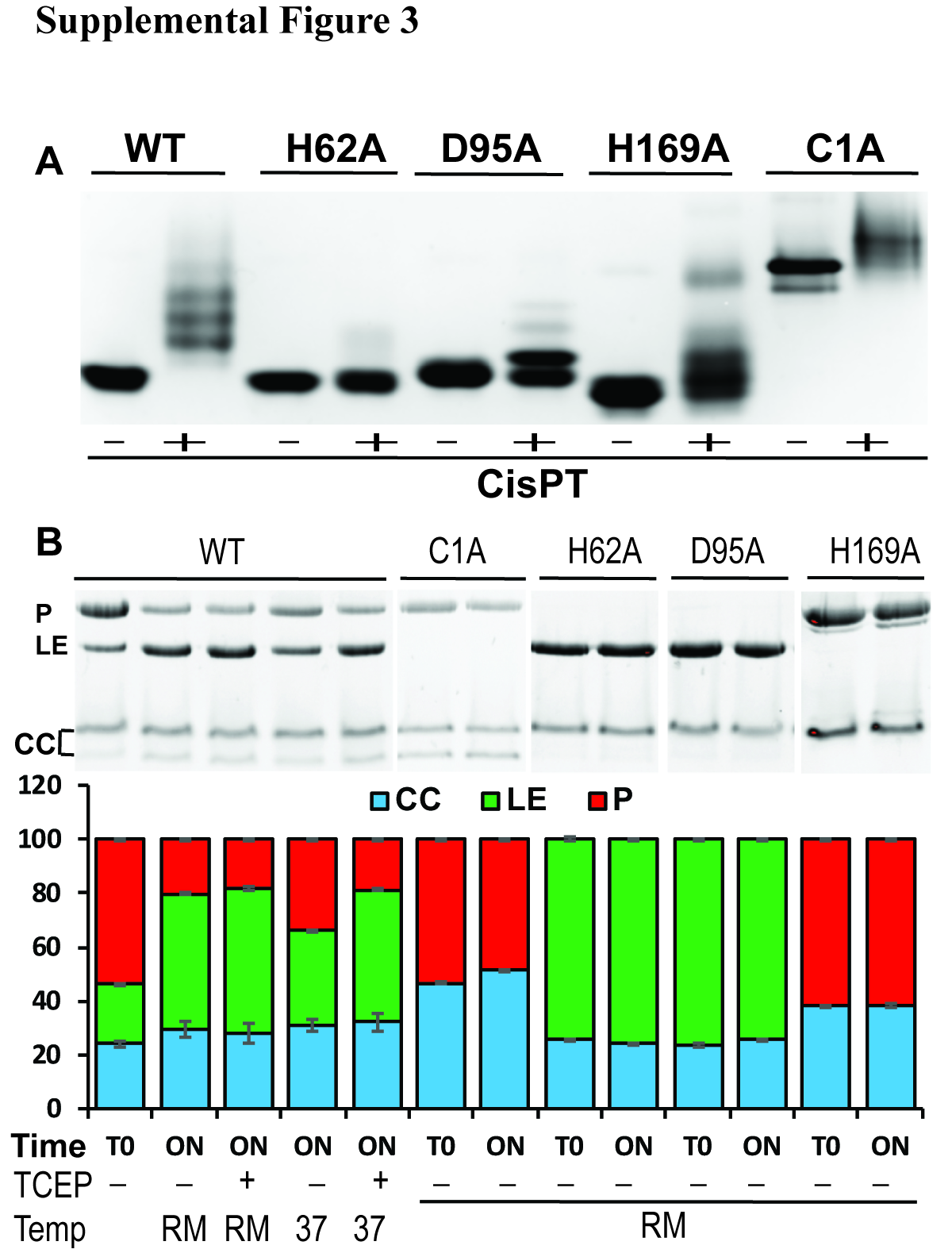
**

**Supplemental Figure 3.** Impact of cisplatin-binding residues on cisplatin binding and the Prp8 intein splicing activities. (**A**) Mutations of cisplatin-binding residues of the Prp8 intein led to loss of cisplatin binding. WT and mutant Prp8 (30 µM) in complex with CisPt (300 µM) (no TCEP) was analyzed in native gel. C1A is an uncleaved Prp8 C1A intein mutant fused to chitin-binding domain (CBD). All others are CBD-cleaved Prp8 inteins. (**B**) SDS-PAGE analysis of WT and mutant Prp8 inteins using the MIG construct. Cell lysates were incubated for 18 hours with/without TCEP (2 mM), at room temperature (RM) or 37°C. Mixtures were analyzed on SDS-PAGE and visualized using GFP fluorescence (upper panel). Quantification of each GFP-containing band was shown in a stacked plot in lower panel. Data are representative of three biological replicates; means and standard deviations are shown.

**Mutagenesis studies of cisplatin-binding residues**

To investigate the functional significance of cisplatin-binding to active site residues, we performed mutagenesis studies, using the same pXI construct. Alanine mutants were generated for cisplatin-binding residues Cys1, His62, Asp95 and His169. Except C1A and H169A, the wild-type (WT) and two other mutants were cleaved efficiently from the CBD-fusion constructs upon DTT treatment at 22 °C, resulting in similar migration positions on native gel (**Supplemental Figure 3A**). Interestingly, compared to WT, and the His62 and Asp95 mutants, H169A displayed reduced splicing efficiency upon DTT treatment at 22 °C (Data not shown). Increasing temperature to 37 °C allowed efficient splicing of H169A from the CBD fusion protein. In construct, the C1A mutant is defective in N-terminal splicing. No cleaved C1A mutant could be obtained, even with overnight treatment of the C1A-CBD fusion protein by 500 mM DTT at 37 °C (Data not shown). Therefore, we used the C1A-CBD fusion protein in the following studies. The C1A-CBD fusion protein migrated to a band position higher than those of WT and other mutants with CBD cleaved (**Supplemental Figure 3A**). To evaluate the mutagenesis effects, we used the gel mobility shift assay. As shown in **Supplemental Figure 3A**, mutations of these residues greatly reduced or abolished the binding of cisplatin to these Prp8 mutants. The results indicated that these residues are essential for the binding of cisplatin to the Prp8 intein.

We also evaluated the effects of these mutations on the Prp8 intein splicing activity. The same alanine mutants were generated in MIG. Using the WT construct, we first evaluated the experimental condition by varying the TCEP and temperature. Our results showed that TCEP is not required to trigger the Prp8 intein splicing at 22°C (**Supplemental Figure 3B**), because the MIG construct is spliced in the absence of TCEP as efficiently as in the presence of TCEP. In addition, the Prp8 splicing activity is more efficient at 22°C than at 37°C. The addition of TCEP at 37°C accelerated the splicing to a level comparable to that at 22°C. Since TCEP does not affect MIG splicing, all the MIG mutant experiments were conducted at 22°C in the absence of TCEP. Our results showed that these mutations displayed differential effects on the Prp8 intein splicing activity (**Supplemental Figure 3B**). The H62A and D95A mutations enhanced the Prp8 intein splicing activity, with all the precursors being spliced. Interestingly, one of the two CC intermediate bands was not observed for these two mutants.

In contrast, the C1A and H169A mutations greatly reduced the Prp8 splicing activity (**Supplemental Figure 3B**). No LE product could be observed for these two mutants. In addition, C-terminal cleavage activities were retained as evidenced by the presence of the by-product intermediate CC for both mutants. However, in contrast to C1A which showed two CC bands, H169A only displayed one CC band, which is similar to two other mutants. Nonetheless, our results confirmed the important role of Cys1 and His169 in the Prp8 intein splicing.

## **Supplemental Table S1**. **Data collection and refinement statistics**.

| Structure | Apo Prp8 | | | Prp8-CisPt complex |
| --- | --- | --- | --- | --- |
| Data collection |  |  |  |  |
|  | λ1 | λ2 | λ3 |  |
| Wavelength (Å) | 0.97932 | 0.97918 | 0.95369 | 1.07147 |
| Resolution (Å) | 40.4-2.6 (2.64-2.6) | 40.4-2.6 (2.65-2.6) | 40.4-2.6 (2.65-2.6) | 35.5-3.06 (3.17-3.06) |
| Space group | P 4_1_ 2_1_ 2 | | | |
| Unit cell |  |  |  |  |
| a,b,c (Å) | 73.88 73.88 192.11 | 73.79 73.79 191.90 | 73.81 73.81 191.92 | 74.85 74.85 191.89 |
| α,β,γ (°) | 90 90 90 | 90 90 90 | 90 90 90 | 90 90 90 |
| Total reflections | 232,440 (20,632) | 230,917 (20,159) | 230,172 (20,299) | 61211 (5657) |
| Unique reflections | 17,347 (1,058) | 17,492 (1,138) | 17,366 (1,058) | 10629 (815) |
| Multiplicity | 13.1 (12.3) | 13.1 (12.6) | 13.1 (12.3) | 5.8 (5.7) |
| Completeness (%) | 100 (100) | 100 (100) | 100 (100) | 95.54 (76.10) |
| Mean I/sigma(I) | 60.7 (2.4) | 60.5 (3.0) | 57.1 (2.2) | 21.58 (2.66) |
| R-merge | 0.038 (0.84) | 0.039 (0.86) | 0.04 (0.83) | 0.107 (1.04) |
|  |  |  |  |  |
| MAD phasing |  |  |  |  |
| Riso | 0.056 | 0.053 |  |  |
| Rano | 0.036 | 0.052 | 0.037 |  |
| Se sites | 9  0.35 | | |  |
| Figure-of-merit |  |  |  |  |
|  |  |  |  |  |
| Refinement |  |  |  |  |
| R-work | 0.22 (0.32) |  |  | 0.25 (0.37) |
| R-free | 0.28 (0.38) |  |  | 0.30 (0.46) |
| No. of non-H Atoms | 3449 |  |  | 3453 |
| Macromolecules | 3444 |  |  | 3444 |
| Ligands | 0 |  |  | 9 |
| RMS (bonds) (Å) | 0.002 |  |  | 0.003 |
| RMS (angles) (°) | 0.57 |  |  | 0.84 |
| Ramachandran |  |  |  |  |
| Favored (%) | 96.52 |  |  | 97.26 |
| Allowed (%) | 3.48 |  |  | 2.74 |
| Outliers (%) | 0 |  |  | 0.00 |
| Average B-factor | 78.4 |  |  | 97.47 |
| Macromolecules | 78.4 |  |  | 97.10 |
| Ligands | 67.1 |  |  | 237.32 |

Statistics for the highest-resolution shell are shown in parentheses.

**Supplemental Table 2**. **Primers used in the manuscript**

| RLuc_N_P26_F | gtttaactttaagaaggagatatacat[a](javascript:__.opf('f5072'))tgGCTTCCAAGGTGTACGAC |
| --- | --- |
| RLuc_C_P26_R | GACGGAGCTCGAATTCGGATCCTCACTGCTCGTTCTTCAGCACGC |
| Prp8_N_Rluc229_F | GATCCCTCTCGTTAAGGGAGGCTGTCTGCAGAACGGTACCCG |
| Prp8_addHis_R | CATGATGTGTAACAAGGTACCatggtgatggtgatggtgACCGGCTAATGAAGAAGTGGA |
| Prp8_addHis_F | TCCACTTCTTCATTAGCCGGTcaccatcaccatcaccatGGTACCTTGTTACACATCATG |
| PRP8_C_Rluc231_R | GGACAATCTGGACGACGTCGGcTGAGTTGTGTAATACCAAATAGTCATGACGG |
| Cga_pXI_C1A_F | GAATTCGAGAAAGCCGCGCTGCAGAACGGTACC |
| Cga_pXI_C1A_R | GGTACCGTTCTGCAGCGCGGCTTTCTCGAATTC |
| Cga_H62A_F | GAAGATCTGGTTTGTACAGCGAATCACATCCTTTCACTG |
| Cga_H62A_R | CAGTGAAAGGATGTGATTCGCTGTACAAACCAGATCTTC |
| Cga_D95A_F | GATAGCTATGAGAGAGTTGCGATAACTGTCGATGACTTTG |
| Cga_D95A_R | CAAAGTCATCGACAGTTATCGCAACTCTCTCATAGCTATC |
| Cga_pXI_H169A_F | GTCATGACTATTTGGTATTAGCGAACTAAAAGCTTGGCACTGG |
| Cga_pXI_H169A_R | CCAGTGCCAAGCTTTTAGTTCGCTAATACCAAATAGTCATGAC |
| Cga_MIG_C1A_F | CATTCTGGGAGAAAGCCGCGCTGCAGAACGGTACCC |
| Cga_MIG_C1A_R | GGGTACCGTTCTGCAGCGCGGCTTTCTCCCAGAATG |
| Cga_MIG_H169A_F | GTCATGACTATTTGGTATTAGCGAACTCAGGATTTGAGGAGATC |
| Cga_MIG_H169A_R | GATCTCCTCAAATCCTGAGTTCGCTAATACCAAATAGTCATGAC |
| pXL1_Cgaprp8_N_F | CAGAAAACTTCAAAGGCCGGCCtagccaccatgCATGCATTCTGGGAGAAAGCC |
| cgaPr8_A132_Flag3x_R | *CATGGATCCCAGTGGACCCTGGGCTAATGAAGAAGTGGAAGGCTG* |
| cgaPr8_A132_Flag3x_F | *CAGCCTTCCACTTCTTCATTAGCC*CAGGGTCCACTGGGATCCATG |
| flag3x_Cgaprp8_T133_R | GATAGACATGATGTGTAACAAGGTtccggagccTCCCTTGTCATCATCATCCTTGG |
| flag3x_Cgaprp8_T133_F | CCAAGGATGATGATGACAAGGGAggctccgga*ACCTTGTTACACATCATGTCTATC* |
| pXL1_CgaPr8_C-R | CCATTACTACTGCTACTGTAACCCTTAATTAATCACGACTCCTCAAATCCTGAG |
| IDT4283 | TTGTGGCAGCTTCAAGAATTCGAGAAAGCCTGTCTGCAGAACGGTACC |
|  | *Note: Cga* Prp8 intein pXI Forward EcoRI site, Infusion primers plus 3 native N-extein (EKA) |
| IDT4284 | AACGACGGCCAGTGCCAAGCTTTTAGTTGTGTAATACCAAATAGTCATGACGGAGATAAAGG |
|  | *Note: Cga* Prp8 intein pXI Reverse HindIII site, Infusion primers |
| IDT4038 | CTCCGTCGACAAGCTTcgTGTCTGCAGAACGGTACCCG |
|  | *Note: Cga* Prp8 intein, pET28a Forward, Infusion primers |
| IDT4039 | TGCTCGAGTGCGGCCGCttaTGAGTTGTGTAATACCAAATAGTCATG |
|  | *Note: Cga* Prp8 intein, pET28a Reverse, Infusion primers |
| IDT3977 | gggGCATGCaTTCTGGGAGAAAGCCTGTCTG |
|  | *Note: Cga* Prp8 intein, pACYC Duet MIG primers, SphI end, traditional cloning |
| IDT3978 | gggATCGATCTCCTCAAATCCTGAGTTGTG |
|  | *Note: Cga* Prp8 intein, pACYC Duet MIG primers, ClaI end, traditional cloning |
| IDT3995 | CCTCCAGATCAATAGACATGATGTG |
|  | Note: Reverse SOEing PCR primer to remove internal ClaI site from *Cga* intein |
| IDT3996 | CATGTCTATTGATCTGGAGGAAAAG |
|  | Note: Forward SOEing PCR primer to remove internal ClaI site from *Cga* intein |
